# POCO: Scalable Neural Forecasting through Population Conditioning

**Published:** 2025-06-17

**Authors:** Yu Duan, Hamza Tahir Chaudhry, Misha B. Ahrens, Christopher D. Harvey, Matthew G. Perich, Karl Deisseroth, Kanaka Rajan

**Affiliations:** 1 EECS, MIT; 2 SEAS, Harvard University; 3 Janelia Research Campus; 4 Harvard Medical School; 5 Kempner Institute; 6 Université de Montréal; 7 Mila - Quebec AI Institute; 8 Stanford University

## Abstract

Predicting future neural activity is a core challenge in modeling brain dynamics, with applications ranging from scientific investigation to closed-loop neurotechnology. While recent models of population activity emphasize interpretability and behavioral decoding, neural forecasting—particularly across multi-session, spontaneous recordings—remains underexplored. We introduce POCO, a unified forecasting model that combines a lightweight univariate forecaster with a population-level encoder to capture both neuron-specific and brain-wide dynamics. Trained across five calcium imaging datasets spanning zebrafish, mice, and *C. elegans*, POCO achieves state-of-the-art accuracy at cellular resolution in spontaneous behaviors. After pre-training, POCO rapidly adapts to new recordings with minimal fine-tuning. Notably, POCO’s learned unit embeddings recover biologically meaningful structure—such as brain region clustering—without any anatomical labels. Our comprehensive analysis reveals several key factors influencing performance, including context length, session diversity, and preprocessing. Together, these results position POCO as a scalable and adaptable approach for cross-session neural forecasting and offer actionable insights for future model design. By enabling accurate, generalizable forecasting models of neural dynamics across individuals and species, POCO lays the groundwork for adaptive neurotechnologies and large-scale efforts for neural foundation models. Code is available at https://github.com/yuvenduan/POCO.

## 1 Introduction

The ability to predict future states from the past is a critical benchmark for models of complex systems such as the brain [32]. Models capable of rapidly and accurately forecasting future neural activity across large spatial-temporal scales—rather than merely fitting historical data—are critical for applied technologies such as closed-loop optogenetic control [19]. Here, we focus on a time-series forecasting (TSF) setup: Given a recent history of measured neural activity, can we predict the neural population dynamics in the near future?

Recently, the increasing ability to simultaneously record from large populations of neurons has motivated a wide range of models of neural population dynamics [15, 27, 46, 60]. However, existing work has primarily focused on interpreting features of these high-dimensional dynamics, whereas neural forecasting is relatively under-explored. In addition, previous work has been mostly limited to a handful of brain regions during controlled behavioral tasks, often using short, trial-based data from individual animals. While trial-based data make modeling tractable due to strong behavioral constraints, a comprehensive understanding of neural dynamics benefits from whole-brain recordings during spontaneous, task-free behaviors. Growing evidence for shared neural motifs across individuals [42, 12, 37], along with the rise of large-scale, multi-animal datasets, motivates the development of *foundation models*—models trained across individuals that generalize to unseen subjects [5, 6, 2]. However, classical models of population dynamics predominantly focus on fitting single-session recordings [40, 27, 15], limiting their ability to utilize larger datasets and capture common motifs shared across animals.

To address these gaps, we developed POCO (POpulation-COnditioned forecaster), a unified predictive model for forecasting spontaneous, brain-wide neural activity. Trained on multi-animal calcium imaging datasets, POCO predicts cellular-resolution dynamics up to ~15 seconds into the future. It combines a simple univariate forecaster for individual neuron dynamics with a population encoder that models the influence of global brain state on each neuron, using Feature-wise Linear Modulation (FiLM) [39] to condition forecasts on population-level structure. For the population encoder, we adapt POYO [5]—originally developed for behavioral decoding in primates—to summarize high-dimensional population activity across sessions. We benchmark POCO against standard baselines and state-of-the-art TSF models trained on five datasets from zebrafish, mice, and *C. elegans*.

In sum, our key contributions are: (1) We introduce POCO, a novel architecture that combines a local forecaster with a population encoder, for cellular-level neural dynamics forecasting. (2) We benchmark the forecasting performance of a wide range of models on five diverse calcium imaging datasets spanning different species, with a focus on neural recording during spontaneous behaviors. (3) We demonstrate that POCO scales effectively with longer recordings and additional sessions, and pre-trained POCO can quickly adapt to new sessions. (4) We conduct extensive analyses on factors that affect performance, including context length, dataset pre-processing, multi-dataset training, and similarity between individuals. These analyses provide critical insights for future work on multi-session neural forecasting.

## 2 Related Work

### Neural Foundation Models.

A growing body of work aims to develop unified models trained on neural data across multiple subjects, tasks, and datasets. This foundation model approach has been applied to spiking data in primates [5, 53, 52, 56, 2], human EEG and fMRI recordings [8, 10, 48], and calcium imaging in mice [6]. While much of this work emphasizes improving behavioral decoding performance [5, 6, 53], some studies have explored forward prediction [17, 2, 56, 38]. However, these efforts are largely confined to spiking data recorded during short, trial-based motor or decision-making tasks. Neural prediction has also been explored in *C. elegans* [44], but the setup is limited to next-step prediction using autoregressive models.

### Models of Population Dynamics.

To understand high-dimensional population dynamics, one line of work has focused on inferring low-dimensional latent representations from observations with models including RNNs [15, 35, 40], switching linear dynamical systems [18, 27], sequential variational autoencoder (VAE) [46, 57, 37, 60], and latent diffusion models [22]. While some of these models could, in theory, be adapted for forecasting, their focus to date has been to gain interpretable insights, especially in constrained neuroscience tasks.

### Time-Series Forecasting.

Time series forecasting is a general problem that emerges in a variety of domains [51, 29, 58, 32]. Deep learning has led to a wide range of TSF architectures, including RNNs [43], temporal convolutional networks (TCNs) [28, 33], and Transformer-based models [58, 59, 47]. Simpler models—such as MLP-based architectures [9, 54, 13] and even linear models [49, 55]—often perform competitively or even outperform more complex alternatives. In neuroscience, Zapbench [32] is a recent benchmark for forecasting, though recordings are limited to a single larval zebrafish.

## 3 Method

### 3.1 Problem Setup

In this work, we consider a multi-session TSF problem. For a session j∈[S], we use $x_{t}^{\left(j\right)}\in {\mathbb{R}}^{N_{j}}$ to denote the neural activity at time step t, where $N_{j}$ is the number of neurons recorded in session j. Given population activity of the last C time steps $x_{t-C:t}^{\left(j\right)}:=x_{t-C,\ldots ,t-1}^{\left(j\right)}\in {\mathbb{R}}^{C\times N_{j}}$, we hope to find a predictor f that forecasts the next P steps and minimizes the mean squared error L:

(1) $$f\left(x_{t-C:t}^{\left(j\right)},j\right)={\tilde{x}}_{t:t+P}^{\left(j\right)},L(f)=E_{j,t}\left[\frac{1}{PN_{i}}{\left\|{\tilde{x}}_{t:t+P}^{\left(j\right)}-x_{t:t+P}^{\left(j\right)}\right\|}_{F}^{2}\right],$$

where we use $\|\cdot {\|}_{F}$ denotes Frobenius norm. In most experiments, we use C=48 and P=16. Importantly, the number of neurons $N_{j}$ varies across sessions, and neurons in different animals do not have one-to-one correspondences; yet, the forecasting problems for different sessions are closely linked due to the similarity in neural dynamics between animals [5, 6, 2], which distinguishes this setting from standard multivariate TSF setups.

### 3.2 Population-Conditioned Forecaster

**Figure 1:**
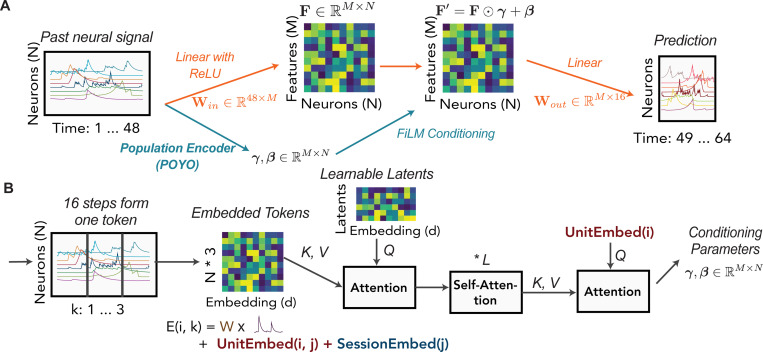
Model Architecture. (A) POCO combines a univariate MLP forecaster (orange part) and a population encoder that conditions the MLP (blue part). This is a schematic for illustration only; traces and feature maps shown are not actual model input, output, or embedding. (B) The population encoder is adapted from POYO [5]. We split the trace of each neuron into several tokens, encode the tokens with POYO, and then use unit embedding to query the conditioning parameters. See the Method section for more details.

We first introduce the overall framework of POCO (Figure 1A). Consider an MLP forecaster with hidden size M=1024 that takes past population activity $x_{t-C:t}^{\left(j\right)}\in {\mathbb{R}}^{C\times N_{j}}$ as input:

(2) $$f_{\text{MLP}}\left(x_{t-C:t}^{\left(j\right)}\right)=W_{out}\text{ReLU}\left(W_{in}x_{t-C:t}^{\left(j\right)}+b_{in}\right)+b_{out},$$

where $W_{in}\in {\mathbb{R}}^{M\times C}$ and $W_{out}\in {\mathbb{R}}^{P\times M}$ are weight matrices. The MLP forecaster is *univariate*, meaning that the prediction for a neuron only depends on its own history, capturing individual auto-correlative properties and simple temporal patterns. Prior work has shown that these simple univariate forecasters perform surprisingly well even for multivariate data [47, 13, 54, 55].

Building on the MLP forecaster, we add a population encoder g that modulates the prediction of the MLP through Feature-wise Linear Modulation (FiLM) [39]. Specifically, the population encoder gives the conditioning parameters $g\left(x_{t-C:t}^{\left(j\right)},j\right)=(\gamma ,\beta )$, where γ, $\beta \in {\mathbb{R}}^{M\times N_{j}}$ are of the same shape as the hidden activations in MLP. We then define POCO as

(3) $$f_{\text{POCO}}\left(x_{t-C:t}^{\left(j\right)}\right)=W_{out}\left[\mathrm{ReLU}\left(W_{in}x_{t-C:t}^{\left(j\right)}+b_{in}\right)⊙\gamma +\beta \right]+b_{out},$$

where ⊙ denotes element-wise multiplication. Intuitively, the FiLM conditioning allows the population encoder to modulate how each neuron’s past activity is interpreted, effectively tailoring the MLP forecaster to the broader brain state at each time point. This enables the model to account for context-dependent dynamics while maintaining neuron-specific predictions.

### 3.3 Population Encoder

We then need to define a population encoder g capable of modeling how the population state influences each neuron. To this end, we adapt a recent architecture, POYO [5, 6], which combines the Perceiver-IO architecture [21] and a tokenization scheme for neural data (Figure 1B). Specifically, for each neuron i, we partition the trace into segments of length $T_{C}=16$ and each segment forms a token, creating $C/T_{C}=3$ tokens for each neuron. Then for each token $k\in \left[C/T_{C}\right]$ of each neuron $i\in \left[N_{j}\right]$, we define the embedding $E(i,k)\in {\mathbb{R}}^{d}$ as

(4) $$E(i,k)=Wx_{r_{k}-T_{C}:r_{k},i}^{\left(j\right)}+b+\text{UnitEmbed}(i,j)+\text{SessionEmbed}(j),$$

where $W\in {\mathbb{R}}^{d\times T_{C}}$ is a linear projection, $r_{k}=t-C+kT_{C}$ defines the temporal boundary of a token, and both UnitEmbed (i,j) and SessionEmbed $\left(j\right)$ are learnable embeddings in ${\mathbb{R}}^{d}$. Intuitively, after learning, the unit embedding can define the dynamical or functional properties of the neuron, while the session embedding can account for different recording conditions (e.g., sampling rates, raw fluorescence magnitude) in different sessions. These token embeddings are arranged into a matrix $E\in {\mathbb{R}}^{CN_{j}/T_{C}\times d}$, which is then processed by Perceiver-IO [21]. Specifically, we use $N_{L}=8$ learnable latents $L_{0}\in {\mathbb{R}}^{N_{L}\times d}$ as the query for the first layer. After L self-attention layers, the final attention layer uses the unit embedding $U_{j}=\text{UnitEmbed}(*,j)\in {\mathbb{R}}^{N_{j}\times d}$ as queries to extract conditioning parameters (γ,β):

(5) $$\begin{array}{l} L_{1}={\mathrm{Attention}}_{0}\left(Q=L_{0};K,V=E\right)\in {\mathbb{R}}^{N_{L}\times d},\\ L_{l+1}={\mathrm{Attention}}_{l}\left(Q,K,V=L_{l}\right)\in {\mathbb{R}}^{N_{L}\times d},l\in [L],\\ L_{L+2}={\text{Attention}}_{L+1}\left(Q=U_{j};K,V=L_{L+1}\right)\in {\mathbb{R}}^{N_{j}\times d},\\ \;\beta =W_{\beta }L_{L+2}^{T}+b_{\beta },\gamma =W_{\gamma }L_{L+2}^{T}+b_{\gamma }, \end{array}$$

where Attention (Q,K,V) is a multi-head attention layer [50], $W_{\gamma }$, $W_{\beta }\in {\mathbb{R}}^{M\times d}$ are learned weight matrices. We have L+2 attention layers in total, where L=1 in most experiments. Following POYO, we use rotary position embedding [45] (details are omitted above for simplicity). One advantage of the Perceiver-IO architecture is that the time complexity only scales linearly with the number of neurons, allowing the model to efficiently scale to recordings of large neural populations. Although we refer to individual neurons throughout, the same framework can also be applied to reduced representations of neural activity, such as principal components (PCs).

Our population encoder has two notable differences from the recent POYO+ model, which is also designed for calcium data [6]. First, POYO+ is designed for behavioral decoding and thus the unit embedding is only used for tokenization. In contrast, here, unit embedding is reused in the last layer to query how the population drives each neuron. Second, in POYO+, $T_{C}$ is always fixed to 1, which creates a massive number of tokens when the context length C and the number of neurons $N_{j}$ are large. We discuss the effect of $T_{C}$ in Figure S15.

## 4 Benchmark

### 4.1 Datasets

To comprehensively test the predictive capability of the models, we used five different datasets from different labs (Table 1). Most recordings were collected during task-free spontaneous behavior, though the Ahrens zebrafish dataset involves responses to visual stimuli [11]. Details of the segmentation pipelines used to extract fluorescence traces are described in the original dataset publications. We z-scored all fluorescence traces to zero mean and unit variance. For experiments involving predicting PCs, we computed PCs after z-scoring individual neurons, and the magnitudes of PCs were preserved. We first cut each session into 1K-step segments, then partitioned each segment into training, validation, and test sets by 3:1:1. See the Appendix for more details.

### 4.2 Baselines

We compared POCO against a diverse set of baselines, from basic linear and auto-regressive models to state-of-the-art methods for time-series forecasting and dynamical system reconstruction. For all models, we used AdamW [30] optimizer with learning rate 0.0003 and weight decay 10^−4^. (1) **MLP** is POCO model without conditioning (Equation 2). (2) **NLinear, DLinear**[55] are variants of univariate linear models that use a linear projection from the context to predictions. (3) **Latent_PLRNN** uses piece-wise linear RNN to model underlying dynamical states linked to observations through linear projection [24, 35]. (4) **TSMixer** [16] is an all-MLP architecture for TSF based on mixing modules for the time and feature dimensions. (5) **TexFilter** [54] learns context-dependent frequency filters for time-series processing. (6) **AR_Transformer** is a basic autoregressive Transformer [50]. (7) **Netformer** [31] infers inter-neuron connection strength via an attention layer, learning a dynamic interaction graph. Here, we add softmax to attention weights for more stable training for multi-step prediction. (8) **TCN** denotes ModernTCN [33], a recent multivariate pure-convolution architecture for TSF. More details about architectures and training can be found in the Appendix.

### 4.3 Copy Baseline and Prediction Score

Lastly, we considered a naive baseline that copies the last observation

(6) $$f_{copy}\left(x_{t-C:t}^{\left(i\right)},i\right)=\left[x_{t-1}^{\left(i\right)},x_{t-1}^{\left(i\right)},\ldots \right],\;\text{(repeat for}\;P=16\;\text{steps)}.$$

Although extremely simple, due to the slow dynamics of calcium traces, $f_{copy}$ is a strong baseline, especially in short-term forecasting [44]. As a more intuitive metric than the raw MSE loss, we define the prediction score as the relative performance improvement compared to the copy baseline, i.e.,

(7) $$\text{Prediction Score}\left(f_{model}\right)=1-L\left(f_{model}\right)/L\left(f_{copy}\right),$$

which is similar to $R^{2}$, but we use the last time step to replace the sample mean.

## 5 Results

### Multi-Session POCO Outperforms Baselines.

**Figure 2:**
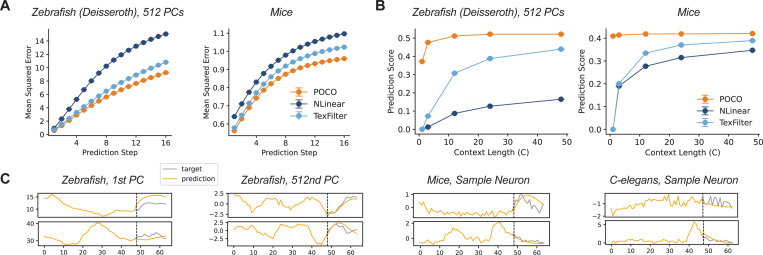
POCO maintains accuracy advantage over time and benefits from longer context. (A) MSE increases when forecasting longer into the future. Results are shown for two different datasets, see Figure S6 for additional datasets. Error bars show SEM of 3 random seeds. (B) Model performance improves with longer context. (C) Sample prediction traces produced by POCO, where the first C=48 steps are given to the model as context.

We tested POCO against baselines on five calcium imaging datasets (Table 2). Sample prediction traces for POCO are shown in Figure 2C. For zebrafish datasets, we first considered the more tractable problem of predicting the first 512 principal components (PCs) due to the large number of neurons. We compared training a different model for each session (single-session models, SS) and training a shared model for all sessions (multi-session models, MS). POCO consistently benefited from multi-session training, outperforming all baselines on four out of five datasets. Other models—such as PLRNN, TexFilter, and NetFormer—also show performance gains from multi-session training. We obtained similar results measuring prediction errors by MSE and MAE (mean absolute error), as shown in Table S6 and S7.

We further evaluated a subset of efficient multi-session models on two zebrafish datasets at single-cell resolution (Table 3). Multi-session POCO again outperformed all baselines, demonstrating its effectiveness in modeling both original neural activity and PCA-reduced activity.

### Effect of Context and Prediction Length.

We observed that prediction error gradually increases over P=16 prediction steps, but POCO generated relatively accurate predictions across all time steps (Figure 2A). To evaluate the impact of context length, we also tested on shorter context lengths C∈{1,3,12,24}, and adjusted POCO’s token length $T_{C}$ to {1,1,3,6} to control the number of tokens. We found that univariate models perform poorly with short contexts, consistent with results in recent work [32]. POCO outperformed baselines in different context lengths (Figure 2B). The prediction accuracy increased with context length but plateaus beyond C>12, suggesting that most of the predictive information is contained within a relatively short temporal window.

### POCO Performance Improvements Scale with Recording Length.

**Figure 3:**
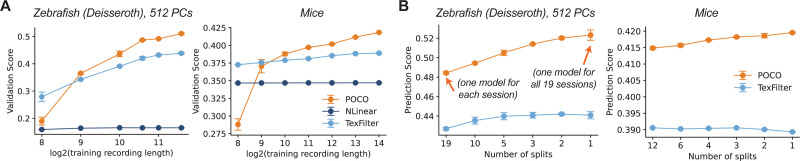
POCO performance improves with longer recordings and more sessions. (A) Prediction score vs. training recording length (x-axis in log scale) for two different datasets. Models were trained using increasing portions of each session’s data. Error bars show SEM across 3 random seeds. (B) We split all sessions in one dataset into n approximately equal partitions and train one model on each partition, then take the average of model prediction scores across all partitions. Average prediction scores vs the number of splits n is shown for two datasets.

We next tested how POCO performance scales with dataset size. First, instead of using the full training partition in each session, we tested the model performance when only the first $T_{train}$ steps are used (Figure 3A, S7). We found that POCO shows steady improvement when longer recordings are used for training, whereas TexFilter shows slower improvements, and NLinear shows no apparent improvement. Second, we split sessions in one dataset into several approximately even partitions and train one model for each partition (Figure 3B, S8). We found that POCO consistently benefits from training on more sessions. Taken together, these results suggest that POCO effectively leverages longer recordings across sessions to learn complex neural dynamics.

### POCO Does Not Consistently Benefit from Multi-Species Training.

To see if datasets from multiple species improves performance, we trained POCO on different datasets simultaneously. Specifically, in each model update step, we aggregated the loss computed from one random batch of data in each dataset. We found that the model generally does not benefit from multi-species training (Table 4). This may be due to differences across datasets in pre-processing pipelines, recording conditions, and, perhaps more importantly, differences in the underlying neural dynamics of recorded species, animals’ behavioral states, and specific brain regions (Table 1). Given this result and a recent report that pre-training on other specimens does not improve neural forecasting performance in zebrafish [20], we hypothesize that the model performance only significantly benefits from more sessions when the modeled systems are *similar enough*. We explored this hypothesis in the following simulation experiment.

### Simulation.

**Figure 4:**
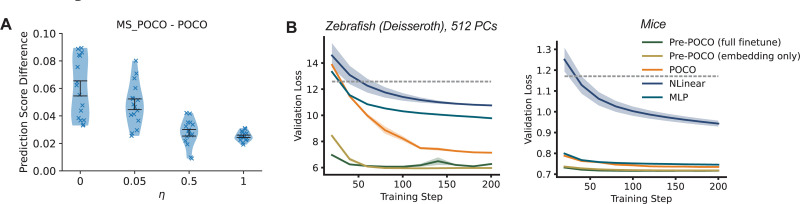
Multi-session POCO improves when individuals are similar; POCO can quickly adapt to new sessions (A) Performance gain of multi-session POCO compared to single-session POCO on synthetic data for different values of η. Larger η means individuals are less similar. We randomly generated 16 cohorts, each with 16 individuals. Each blue cross represents a cohort. Error bars are SEM. (B) Validation loss curve of fine-tuning pre-trained POCO (Pre-POCO) and training POCO, NLinear, and MLP from scratch. We also compared full-finetuning with only tuning the embedding. Dashed gray lines represent the copy baseline. Error shades represent SEM for 3 random seeds. Two sample sessions from two datasets are shown here; see Figure S12 for more sessions.

Here, we used simulated data to test how similarity between individuals influences multi-session model performance. To generate neural data from a synthesized cohort, we first randomly sample a template connectivity matrix $J_{0}∼N(0,I)\in {\mathbb{R}}^{n\times n}$, where we use n=300 neurons. Then for each synthesized individual i∈ [16], we set

$$J_{i}=\sqrt{1-\eta^{2}}J_{0}+\eta \epsilon_{i},\epsilon_{i}∼N(0,I)$$

where $\epsilon_{i}$ is random deviation from the template connectivity matrix, η∈[0,1] controls the similarity between individuals. We set the coefficient for $J_{0}$ to be $\sqrt{1-\eta^{2}}$ so that $J_{i}$ keeps unit variance. We then used each $J_{i}$ as the connectivity of a noisy spontaneous RNN to generate synthetic neural data (see the Appendix for details) and trained POCO as on the multi-session neural data above (Figure 4A). POCO showed greater benefit from multi-session training when individuals shared similar connectivity patterns (η≤0.05), compared to when their connectivity was independent (η=1).

We also compared POCO with baselines on single-session simulation data. Surprisingly, models including PLRNN, auto-regressive Transformer, and TSMixer performed significantly better than POCO, despite their relatively poor performance on real neural datasets (Figure S11). Real neural data likely exhibits greater non-stationarity, multi-scale dependencies, and heterogeneous noise profiles than our current simulations, properties which POCO’s architecture may be better suited to handle than models excelling in the simulated regime.

### Finetuning on New Sessions.

A core benefit of the foundation model approach is that it allows rapid adaptation to new sessions. We pre-trained POCO on 80% of sessions and fine-tuned this model on each of the remaining ones (see the Appendix for details). For finetuning, we compared full finetuning with only finetuning the unit and session embedding. We found that pre-trained models achieved reasonable predictive performance in only tens of training steps (Figure 4B). In addition, full finetuning leads to limited or no improvements compared to only fine-tuning the unit embedding (Table S9). Rapid adaptation is crucial for real-time, closed-loop applications: in our setup, fine-tuning the embedding for 200 training steps takes less than 15 seconds, and the forecasting inference time is only 3.5 ms (see the Appendix for details). Consistent with previous results on multi-dataset training, pre-training on different datasets does not improve performance (Table S10).

### Analyzing Unit Embedding.

**Figure 5:**
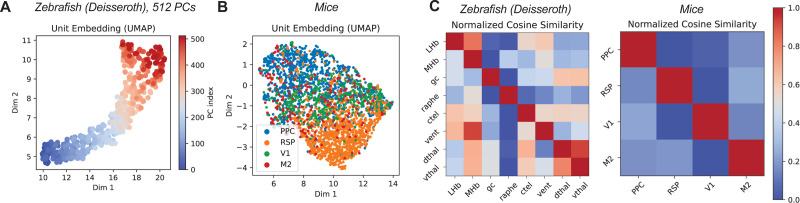
POCO learns meaningful unit embeddings without supervision. (A) UMAP [34] visualization of unit embeddings after training POCO on the first 512 PCs in a zebrafish dataset. (B) Visualization of unit embedding after training POCO on the mice dataset, where neurons are colored by the brain region. One sample session is shown for (A) and (B), see Figure S9 for more sessions. (C) Normalized average cosine similarity of unit embeddings between each pair of regions. Each row is normalized to [0, 1] and then averaged across 4 runs. Patterns are consistent for different seeds (Figure S10). See the Appendix for more details.

Recent work shows that unit embeddings in POYO learns region and cell-type-specific information when trained for behavioral decoding [6]. We analyzed the trained multi-session POCO model to determine if similar structure emerges. We found that when trained on PCs, unit embeddings are consistently distributed according to the order of the PCs (Figure 5A). When trained on neurons, we found significant clustering of neurons by brain region, as indicated by the average pair-wise cosine similarity (Figure 5C). In particular, retrosplenial cortex (RSP) neurons in mice form a particularly distinct cluster (Figure 5B). Thus, POCO learns to encode functional dynamical properties in the unit embedding when trained for forecasting, even when no prior knowledge (e.g., neuron location) is given to the model.

### Effect of Filters.

Filtering high or low frequency components is a common preprocessing technique in calcium imaging, used to remove slow drifts or fast noise that are not directly related to neural dynamics [23, 36]. By default, we used no temporal filter to maximally preserve neural signals. However, we found that with low-filtering, POCO still outperforms baselines except on *C. elegans* datasets (Table S8). Low-pass filters improved models’ performance compared to the copy baseline in most datasets, suggesting that high-frequency components are generally harder to predict (Figure S13). Low-pass filtering also helped POCO to benefit more from multi-session training (Figure S8).

### Zapbench Evaluation.

Although the main focus of this work is on multi-session datasets, we also tested our model on a recent neural population forecasting benchmark, Zapbench [32], which contains light-sheet microscopy recordings of 71721 neurons over 7879 time steps for one zebrafish. We followed the setup in Zapbench to partition the dataset and evaluate our model with short (C=4) or long (C=256) context, and prediction length P=32. We found that when C=4, POCO outperforms other trace-based methods and performs comparably to UNet, a computationally expensive model operating directly on raw volumetric videos rather than segmented neural traces [20]. At longer contexts (C=256), POCO underperforms relative to UNet (Figure S14). See the Appendix for more details.

### Ablation Study.

To test the necessity of the components of POCO, we removed or replaced some parts of the model and compared performance. Specifically, we tested (1) directly using the POYO model to generate 16-steps prediction instead of generating conditioning parameters (POYO only); (2) MLP without conditioning; and (3) MLP conditioned by a univariate Transformer that takes the calcium trace of a single neuron instead of encoding the whole population (see the Appendix for more details). Both the MLP forecaster and the population encoder were necessary for full performance (Table 5). We also tested how key model hyperparameters influence performance, including the length of the token (Figure S15), embedding dimension (Figure S16), number of layers (Figure S17), the number of latents (Figure S18), learning rate (Figure S19) and weight decay (Figure S20). We found that POCO performance is relatively stable for a range of hyperparameter settings, and even a small POYO encoder is sufficient.

## 6 Discussion

In this work, we introduced POCO, a population-conditioned forecaster that combines a local univariate predictor with a global population encoder to capture both neuron-level dynamics and shared brain-state structure. Across five calcium-imaging datasets in zebrafish, mice, and *C. elegans*, POCO achieves state-of-the-art overall forecasting accuracy. Beyond raw performance, we show that POCO rapidly adapts to new sessions with only tens of fine-tuning steps of its embeddings, making it feasible for real-time adaptation during live recordings. Our analysis of POCO’s learned unit embeddings demonstrates that the model autonomously uncovers meaningful population structure such as brain regions, even though no anatomical labels were provided. These findings underscore POCO’s dual strength in accurate prediction and in learning interpretable representations of units. Finally, our results extend the recent progress on scaling up models for behavioral decoding [5, 6] to the realm of neural prediction on spontaneous neural recordings in different species.

We note a few limitations that can be opportunities for future research. First, our results indicate that factors such as calcium indicator dynamics, preprocessing pipelines, and species differences can significantly affect model performance, yet a systematic understanding of their influence remains lacking. Second, our findings highlight the difficulty of multi-dataset and multi-species training—a challenge that may be mitigated by improved architectures or alignment strategies. Third, while POCO learns biologically meaningful unit embeddings within datasets, it is unclear whether these representations are comparable across species or generalize to unseen brain regions. Finally, while we focus on calcium imaging during spontaneous behavior, extending POCO to spiking data and standardized behavioral tasks could enable the use of larger datasets and support modeling of neural dynamics in more structured settings [14, 26].

## Supplementary Material

Supplement 1

## Figures and Tables

**Table 1:** Overview of the five datasets. $f_{s}$ is the sampling frequency. The number of neurons, recording length, and sampling frequency vary by session, the approximate average is shown here.

Species	Lab	#Sessions	#Neurons	#Steps	$f_{s}$	Ca^2+^ Indicator
Larval Zebrafish	Deisseroth[1]	19	11K	4.3K	1.1Hz	GCaMP6s
Larval Zebrafish	Ahrens[11]	15	77K	3.9K	2.1Hz	GCaMP6f
Mice	Harvey[40, 3]	12	1.6K	15K	5.4Hz	GCaMP6s
*C. elegans*	Zimmer[23]	5	126	3.1K	2.8Hz	GCaMP5K
*C. elegans*	Flavell[4]	40	136	1.6K	1.7Hz	GCaMP7f

**Table 2:** POCO achieves highest prediction scores across species and datasets. Prediction scores across five datasets show that POCO consistently outperforms baselines, especially in the multi-session setting. Zebrafish data are reduced to 512 PCs. 95% CI from 4 seeds.

	Zebrafish, 512 PCs		Mice	C-elegans	
Model	Deisseroth	Ahrens		Zimmer	Flavell
*Single-Session Models*					
**POCO**	0.466 _±0.019_	**0.433**_**±0.008**_	0.415 _±0.001_	0.329 _±0.009_	0.079 _±0.017_
MLP	0.399 _±0.001_	0.388 _±0.001_	0.409 _±0.000_	0.336 _±0.001_	0.236 _±0.002_
NLinear	0.167 _±0.000_	0.211 _±0.000_	0.348 _±0.000_	0.250 _±0.000_	0.217 _±0.001_
Latent_PLRNN	0.064 _±0.024_	0.212 _±0.002_	0.335 _±0.001_	0.143 _±0.015_	0.170 _±0.005_
TexFilter	0.419 _±0.006_	0.378 _±0.003_	0.389 _±0.000_	0.333 _±0.005_	0.230 _±0.002_
NetFormer	0.204 _±0.013_	0.208 _±0.008_	0.329 _±0.000_	0.145 _±0.004_	0.168 _±0.002_
AR_Transformer	−0.875 _±0.027_	−0.054 _±0.005_	0.312 _±0.005_	−0.320 _±0.063_	−1.024 _±0.038_
DLinear	0.211 _±0.000_	0.290 _±0.000_	0.394 _±0.000_	0.267 _±0.000_	0.221 _±0.000_
TCN	0.153 _±0.010_	0.240 _±0.004_	0.360 _±0.000_	0.305 _±0.003_	0.226 _±0.004_
TSMixer	−0.550 _±0.036_	0.016 _±0.009_	0.390 _±0.001_	0.129 _±0.012_	−0.199 _±0.039_

*Multi-Session Models*					
**MS_POCO**	**0.525**_**±0.004**_	**0.440**_**±0.003**_	**0.420**_**±0.002**_	**0.364**_**±0.005**_	0.213 _±0.030_
MS_MLP	0.417 _±0.002_	0.370 _±0.001_	0.409 _±0.000_	0.348 _±0.002_	**0.274**_**±0.004**_
MS_NLinear	0.165 _±0.000_	0.202 _±0.000_	0.347 _±0.000_	0.253 _±0.000_	0.221 _±0.000_
MS_Latent_PLRNN	0.149 _±0.002_	0.248 _±0.007_	0.355 _±0.000_	0.118 _±0.011_	0.183 _±0.006_
MS_TexFilter	0.440 _±0.005_	0.349 _±0.000_	0.389 _±0.000_	0.346 _±0.000_	0.256 _±0.002_
MS_NetFormer	0.221 _±0.008_	0.220 _±0.002_	0.331 _±0.000_	0.150 _±0.002_	0.217 _±0.001_
MS_AR_Transformer	−0.777 _±0.005_	0.002 _±0.012_	0.317 _±0.003_	−0.333 _±0.043_	−0.675 _±0.022_

**Table 3:** POCO outperforms baselines at single-cell resolution in zebrafish. Prediction scores on raw neural traces (not PCA-reduced) from Ahrens and Deisseroth datasets. All models are multi-session. 95% CI from 4 seeds.

Model	Zebrafish (Ahrens)	Zebrafish (Deisseroth)
**MS_POCO**	**0.429**_**±0.003**_	**0.251**_**±0.004**_
MS_MLP	0.417 _±0.001_	**0.254**_**±0.001**_
MS_NLinear	0.367 _±0.000_	0.172 _±0.001_
MS_TexFilter	0.398 _±0.001_	0.232 _±0.003_

**Table 4:** POCO benefits from multi-session, but not multi-species, training. Comparing single-session, multi-session, multi-species, and zebrafish-only POCO variants. Within-species training yields the best performance. 95% CI from 4 seeds.

	Zebrafish, 512 PCs		Mice	C-elegans	
Model	Deisseroth	Ahrens		Zimmer	Flavell
Single-Session POCO	0.466 _±0.019_	0.433 _±0.008_	0.415 _±0.001_	0.329 _±0.009_	0.079 _±0.017_
Multi-Session POCO	**0.525**_**±0.004**_	**0.440**_**±0.003**_	**0.420**_**±0.002**_	**0.364**_**±0.005**_	0.213 _±0.030_
Multi-Species POCO	0.499 _±0.003_	**0.441**_**±0.003**_	0.403 _±0.000_	0.330 _±0.009_	**0.252**_**±0.011**_
Zebrafish POCO	0.500 _±0.005_	**0.442**_**±0.004**_			

**Table 5:** Both MLP forecaster and population encoder are necessary for POCO. Ablation study shows performance drops when either component is removed or simplified. 95% CI from 4 seeds.

	Zebrafish, 512 PCs		Mice
Model	Deisseroth	Ahrens	
**Full POCO**	**0.525**_**±0.004**_	**0.440**_**±0.003**_	**0.420**_**±0.002**_
POYO only	−0.971 _±0.015_	−0.057 _±0.001_	0.332 _±0.001_
MLP only	0.417 _±0.002_	0.370 _±0.001_	0.409 _±0.000_
MLP conditioned by univariate Transformer	0.463 _±0.001_	0.408 _±0.002_	0.411 _±0.000_
