# Supplementary material for: POCO: Scalable Neural Forecasting through Population Conditioning
Source: ArXiv. 2025 Jun 17:arXiv:2506.14957v1. Preprint. [Version 1] (PMC12447713)
Supplement: Supplement 1 [file NIHPP2506.14957v1-supplement-1.pdf]

A Supplementary Results

Table S6: **MSE comparison across datasets.** Mean squared error (MSE) across five datasets, using the same model configurations as in Table 2. Different from Table 2 where the score is computed by averaging across all sessions, here sessions are weighted by the size of the test set ($N_i \times |D_{test}|$, where D_{test} is the number of sequences in the test set), so longer recording sessions and sessions with more neurons recorded will receive a larger weight.

Model	Zebrafish, 512 PCs		Mice	C-elegans	
	Deisseroth	Ahrens		Zimmer	Flavell
Single-Session Models					
**POCO**	6.022 ±0.083	**40.118 ±0.473**	0.845 ±0.002	0.369 ±0.005	0.696 ±0.011
MLP	7.082 ±0.021	43.335 ±0.024	0.853 ±0.000	0.365 ±0.001	0.582 ±0.001
NLinear	9.587 ±0.005	55.742 ±0.016	0.944 ±0.000	0.411 ±0.000	0.597 ±0.000
Latent_PLRNN	10.423 ±0.152	55.047 ±0.189	0.964 ±0.001	0.470 ±0.007	0.633 ±0.005
TexFilter	6.590 ±0.066	44.040 ±0.215	0.882 ±0.000	0.368 ±0.003	0.588 ±0.001
NetFormer	8.877 ±0.129	56.169 ±0.527	0.973 ±0.000	0.466 ±0.002	0.635 ±0.001
AR_Transformer	18.929 ±0.095	73.523 ±0.632	1.008 ±0.012	0.688 ±0.032	1.517 ±0.029
DLinear	8.986 ±0.003	49.993 ±0.018	0.876 ±0.000	0.401 ±0.000	0.594 ±0.000
TCN	9.575 ±0.076	53.725 ±0.254	0.926 ±0.001	0.383 ±0.002	0.591 ±0.003
TSMixer	15.516 ±0.354	68.733 ±0.682	0.882 ±0.002	0.468 ±0.004	0.911 ±0.027
Multi-Session Models					
**MS_POCO**	**5.498 ±0.063**	**39.678 ±0.198**	**0.839 ±0.003**	**0.350 ±0.003**	0.600 ±0.023
MS_MLP	6.801 ±0.022	44.612 ±0.069	0.854 ±0.000	0.358 ±0.001	**0.554 ±0.003**
MS_NLinear	9.565 ±0.015	56.477 ±0.006	0.946 ±0.000	0.409 ±0.000	0.594 ±0.000
MS_Latent_PLRNN	9.597 ±0.026	52.571 ±0.435	0.936 ±0.000	0.476 ±0.005	0.621 ±0.004
MS_TexFilter	6.377 ±0.053	46.221 ±0.055	0.882 ±0.001	0.361 ±0.000	0.568 ±0.001
MS_NetFormer	8.803 ±0.076	55.351 ±0.210	0.970 ±0.001	0.461 ±0.001	0.597 ±0.000
MS_AR_Transformer	18.268 ±0.025	70.082 ±0.767	0.997 ±0.004	0.696 ±0.015	1.261 ±0.017

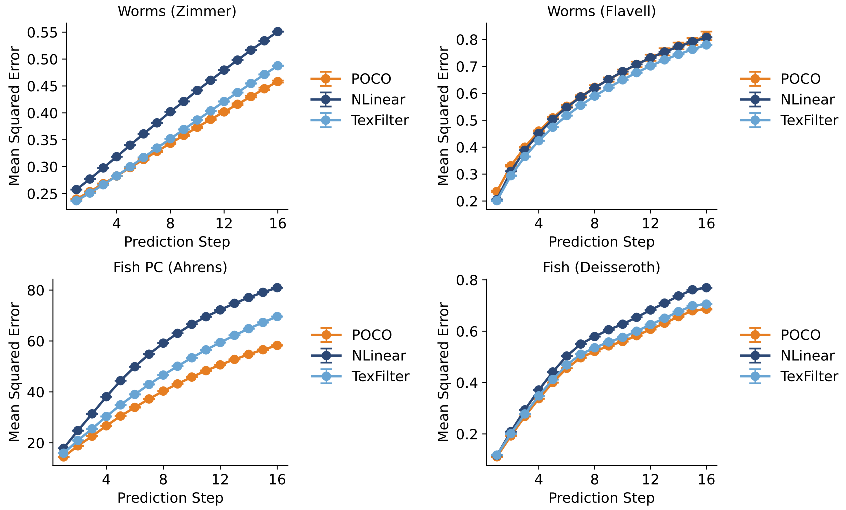

Figure S6: **Forecasting error increases with prediction horizon across datasets.** Mean squared error (MSE) plotted over 16 prediction steps for four additional datasets. POCO maintains better performance over time compared to NLinear and TexFilter. Error bars show SEM across 3 seeds.

Table S7: **Mean absolute error (MAE) comparison across datasets.** Same as Table S6, but reporting mean absolute error (MAE) instead of prediction score. 95% confidence intervals estimated with 4 random seeds.

Model	Zebrafish, 512 PCs Deisseroth	Zebrafish, 512 PCs Ahrens	Mice	C-elegans Zimmer	C-elegans Flavell
Single-Session Models					
**POCO**	1.310 ±0.005	4.116 ±0.006	0.645 ±0.001	0.418 ±0.004	0.604 ±0.007
MLP	1.442 ±0.002	4.322 ±0.001	0.648 ±0.000	0.413 ±0.001	0.540 ±0.001
NLinear	1.818 ±0.000	4.872 ±0.001	0.686 ±0.000	0.438 ±0.000	0.545 ±0.000
Latent_PLRNN	1.965 ±0.009	5.092 ±0.008	0.709 ±0.000	0.484 ±0.004	0.559 ±0.002
TexFilter	1.443 ±0.010	4.416 ±0.012	0.657 ±0.000	0.409 ±0.002	0.537 ±0.001
NetFormer	1.757 ±0.013	4.852 ±0.007	0.693 ±0.000	0.471 ±0.001	0.560 ±0.001
AR_Transformer	2.465 ±0.010	5.500 ±0.007	0.712 ±0.006	0.606 ±0.014	0.941 ±0.009
DLinear	1.761 ±0.000	4.645 ±0.001	0.658 ±0.000	0.440 ±0.000	0.547 ±0.000
TCN	1.847 ±0.016	4.822 ±0.002	0.674 ±0.000	0.421 ±0.001	0.539 ±0.002
TSMixer	2.026 ±0.039	5.021 ±0.035	0.665 ±0.001	0.493 ±0.001	0.702 ±0.010
Multi-Session Models					
**MS_POCO**	**1.262 ±0.004**	**4.096 ±0.006**	**0.643 ±0.002**	**0.402 ±0.003**	0.556 ±0.011
MS_MLP	1.422 ±0.001	4.320 ±0.003	0.648 ±0.001	0.407 ±0.002	**0.524 ±0.002**
MS_NLinear	1.824 ±0.001	4.868 ±0.003	0.687 ±0.000	0.437 ±0.000	0.545 ±0.000
MS_Latent_PLRNN	1.892 ±0.004	4.966 ±0.005	0.694 ±0.000	0.488 ±0.002	0.561 ±0.003
MS_TexFilter	1.418 ±0.011	4.444 ±0.007	0.658 ±0.001	0.404 ±0.000	0.526 ±0.002
MS_NetFormer	1.766 ±0.006	4.849 ±0.004	0.692 ±0.000	0.469 ±0.001	0.544 ±0.000
MS_AR_Transformer	2.454 ±0.003	5.421 ±0.065	0.715 ±0.002	0.615 ±0.007	0.862 ±0.006

Table S8: **Low-pass filtering improves prediction in most datasets.** Performance across five datasets after applying a low-pass filter with a cutoff of $0.1 \times f_s$, where f_s is the sampling frequency. Filtering improves POCO's performance relative to the copy baseline in most settings. Results are averaged across sessions; 95% confidence intervals from 4 seeds.

Model	Zebrafish, 512 PCs Ahrens	Mice	C-elegans Zimmer	C-elegans Flavell
Single-Session Models				
**POCO**	0.60 ± 0.02	0.50 ± 0.00	0.21 ± 0.02	-0.04 ± 0.03
MLP	0.59 ± 0.00	0.50 ± 0.00	0.31 ± 0.00	0.35 ± 0.00
NLinear	0.33 ± 0.00	0.33 ± 0.00	0.16 ± 0.00	0.27 ± 0.00
Latent_PLRNN	0.21 ± 0.00	0.19 ± 0.00	0.13 ± 0.02	0.16 ± 0.01
TexFilter	0.61 ± 0.01	0.50 ± 0.01	0.37 ± 0.01	0.34 ± 0.01
NetFormer	0.27 ± 0.00	0.31 ± 0.00	0.01 ± 0.01	0.09 ± 0.00
AR_Transformer	-0.20 ± 0.02	-0.14 ± 0.01	-1.37 ± 0.13	-1.48 ± 0.03
DLinear	0.39 ± 0.00	0.41 ± 0.00	0.17 ± 0.00	0.26 ± 0.00
TCN	0.32 ± 0.01	0.31 ± 0.01	0.19 ± 0.03	0.21 ± 0.02
TSMixer	-0.05 ± 0.03	0.32 ± 0.00	-0.45 ± 0.03	-0.46 ± 0.06
Multi-Session Models				
**MS_POCO**	**0.65 ± 0.01**	**0.55 ± 0.01**	0.32 ± 0.02	0.13 ± 0.05
MS_MLP	0.57 ± 0.00	0.51 ± 0.00	0.35 ± 0.00	0.40 ± 0.00
MS_NLinear	0.32 ± 0.00	0.35 ± 0.00	0.19 ± 0.00	0.30 ± 0.00
MS_Latent_PLRNN	0.24 ± 0.01	0.21 ± 0.00	0.15 ± 0.01	0.19 ± 0.01
MS_TexFilter	0.60 ± 0.01	0.51 ± 0.01	**0.41 ± 0.01**	**0.42 ± 0.01**
MS_NetFormer	0.31 ± 0.01	0.33 ± 0.00	0.11 ± 0.00	0.23 ± 0.00
MS_AR_Transformer	-0.12 ± 0.01	0.01 ± 0.01	-1.59 ± 0.10	-1.07 ± 0.02

Table S9: **Fine-tuning POCO embeddings enables rapid adaptation.** Test performance when fine-tuning a pre-trained POCO (Pre-POCO) compared to training from scratch. We compare full fine-tuning, embedding-only tuning, and MLP+embedding tuning. Embedding-only tuning achieves comparable performance to full fine-tuning. 95% confidence intervals estimated with 3 seeds.

Model	Zebrafish, 512 PCs Deisseroth	Ahrens	Mice
Pre-POCO (full finetune)	**0.532 ±0.014**	**0.424 ±0.017**	**0.406 ±0.001**
Pre-POCO (embedding only)	**0.539 ±0.008**	**0.392 ±0.062**	**0.405 ±0.001**
Pre-POCO (unit embedding + MLP)	**0.534 ±0.009**	**0.412 ±0.015**	0.405 ±0.000
POCO	0.497 ±0.019	**0.424 ±0.020**	0.404 ±0.001
NLinear	0.153 ±0.001	0.197 ±0.000	0.327 ±0.002
MLP	0.386 ±0.003	0.349 ±0.005	0.395 ±0.000

Table S10: **Pre-training on mismatched datasets hurts performance.** POCO models pre-trained on one zebrafish dataset and fine-tuned on another perform worse than models trained from scratch on the target dataset. Even joint pre-training on both datasets underperforms single-dataset training. These results highlight the importance of within-distribution pre-training. 95% confidence intervals estimated with 3 seeds.

Model	Zebrafish, 512 PCs Deisseroth	Ahrens	Mice
Pre-POCO(Ahrens)	0.464 ±0.005	**0.392 ±0.062**	0.290 ±0.014
Pre-POCO(Deisseroth)	**0.539 ±0.005**	0.349 ±0.031	0.338 ±0.005
Pre-POCO(Both Datasets)	0.517 ±0.015	**0.410 ±0.015**	0.304 ±0.007
POCO	0.497 ±0.019	**0.424 ±0.020**	**0.398 ±0.014**

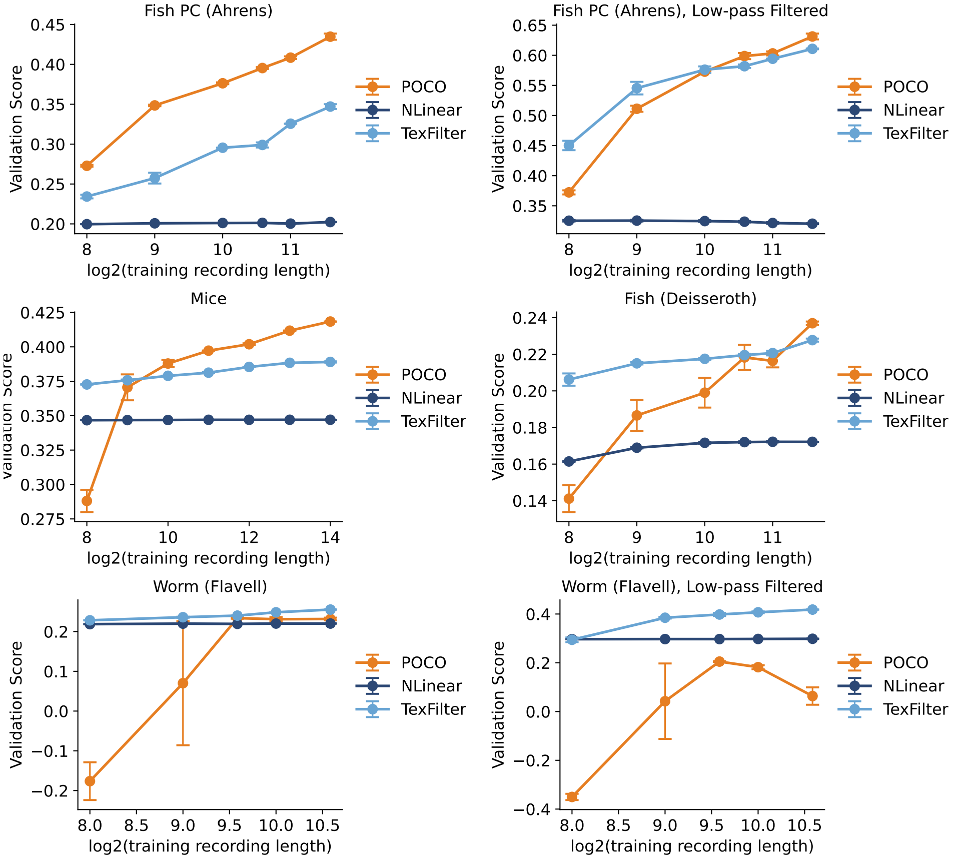

Figure S7: **Longer training recordings improve POCO performance across datasets.** Prediction score increases with log-scaled training duration. Low-pass filtering further enhances gains in some datasets. Error bars show SEM across 2 seeds.

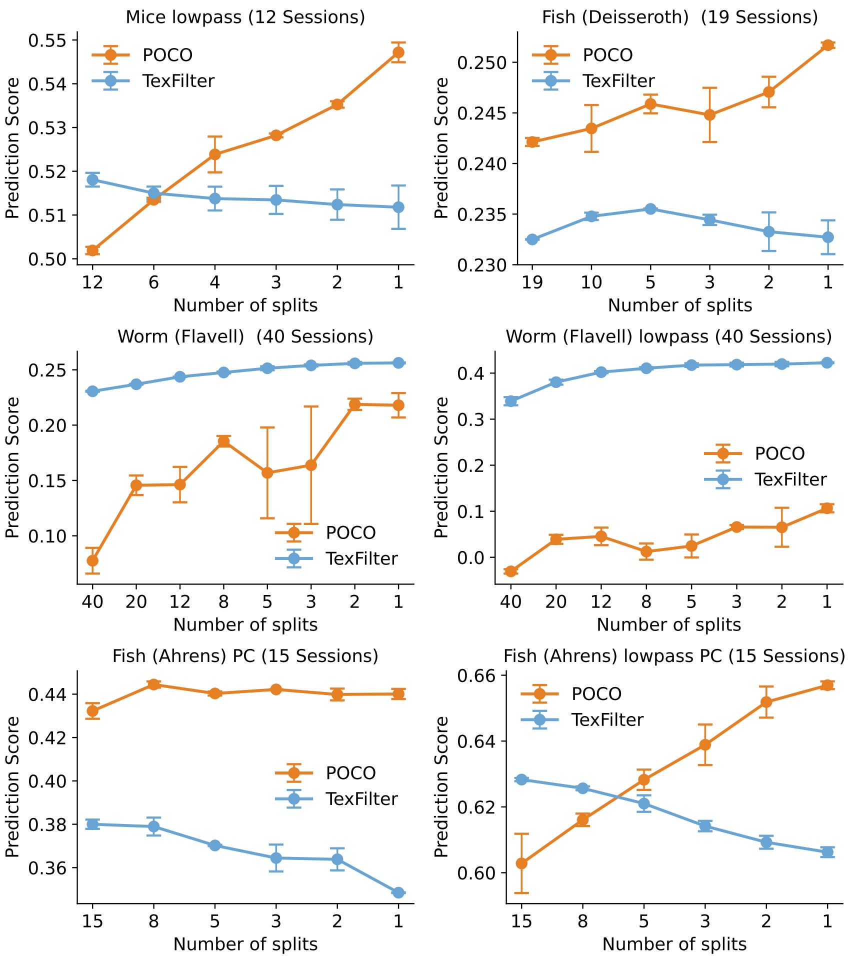

Figure S8: **Multi-session POCO benefits from training on more sessions.** Prediction score improves as more sessions are aggregated. Results are shown for both raw and low-pass filtered datasets. Error bars show SEM across 2 seeds.

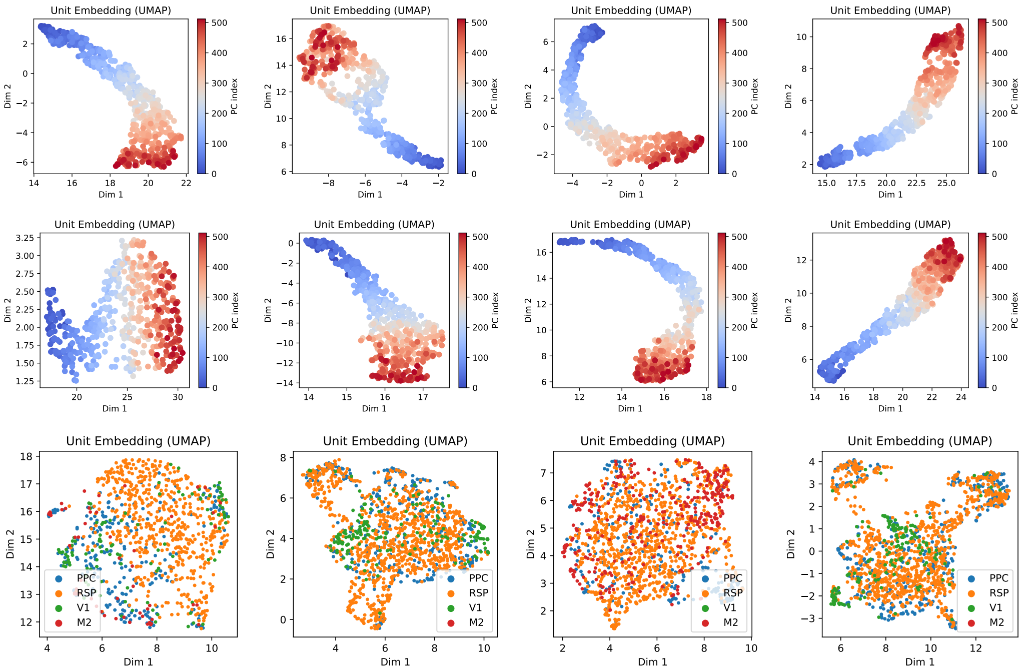

Figure S9: **POCO unit embeddings reflect meaningful structure across sessions.** UMAP projections of unit embeddings from four sessions across zebrafish (Deisseroth and Ahrens) and mouse datasets. Embedding structure is consistent within datasets but varies across sessions.

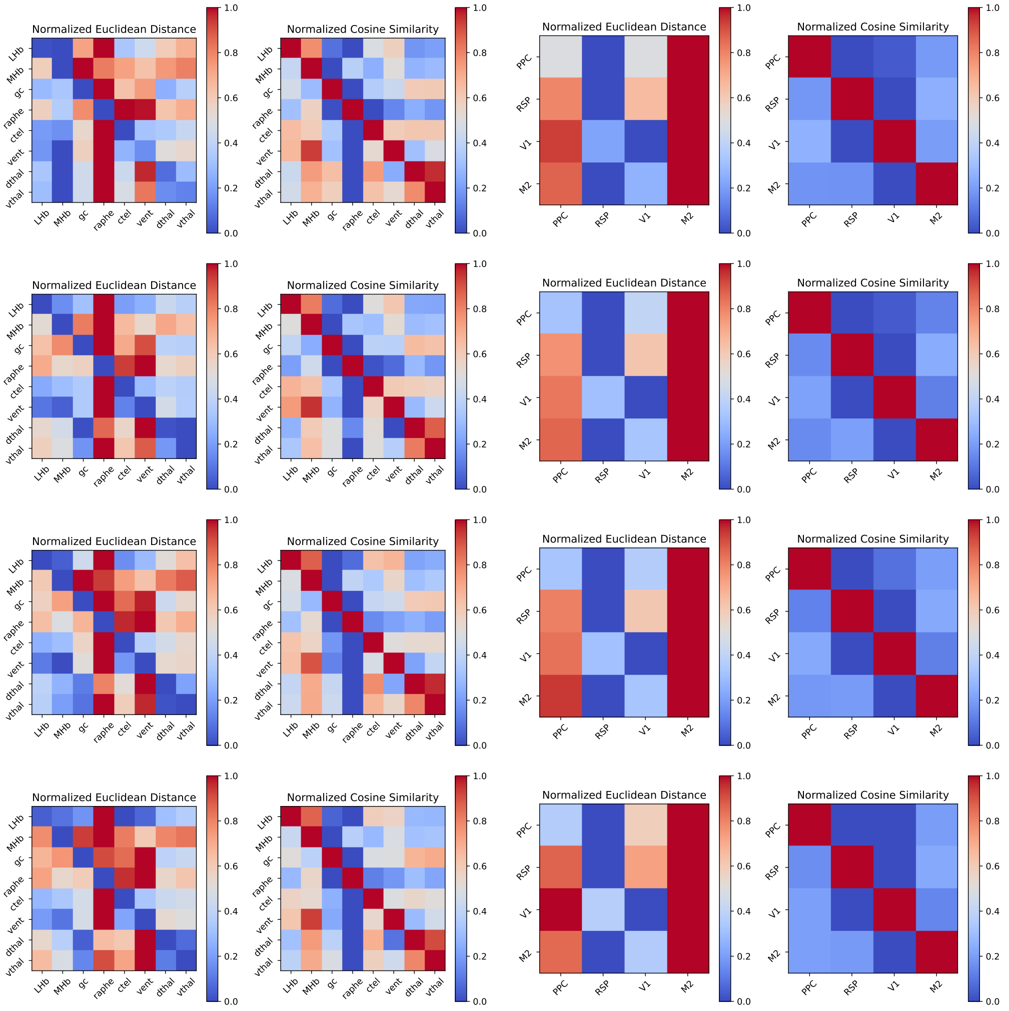

Figure S10: **Unit embeddings cluster by brain region across runs and species.** Normalized cosine similarity and Euclidean distance matrices computed on unit embeddings across zebrafish and mouse sessions. As in Figure 5C, each row is normalized to [0, 1]. From top to bottom are 4 different runs of POCO.

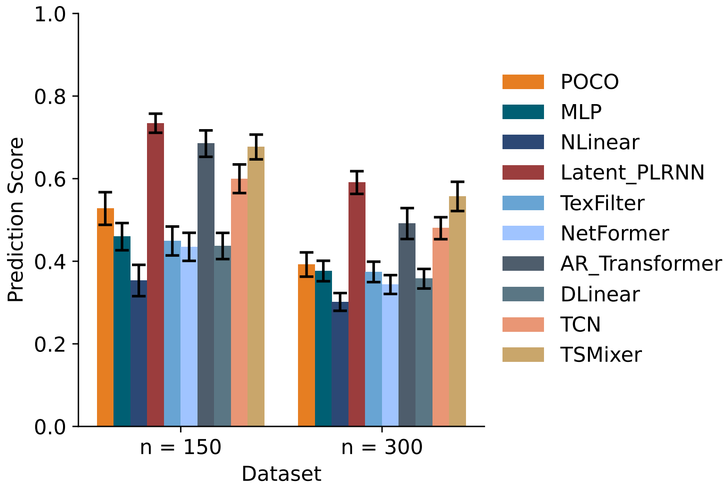

Figure S11: **Baseline models perform well on simulated dynamics.** On single-session synthetic data, PLRNN and TSMixer outperform POCO, unlike on real data. Each point represents a random network instance. Error bars show SEM across 16 seeds.

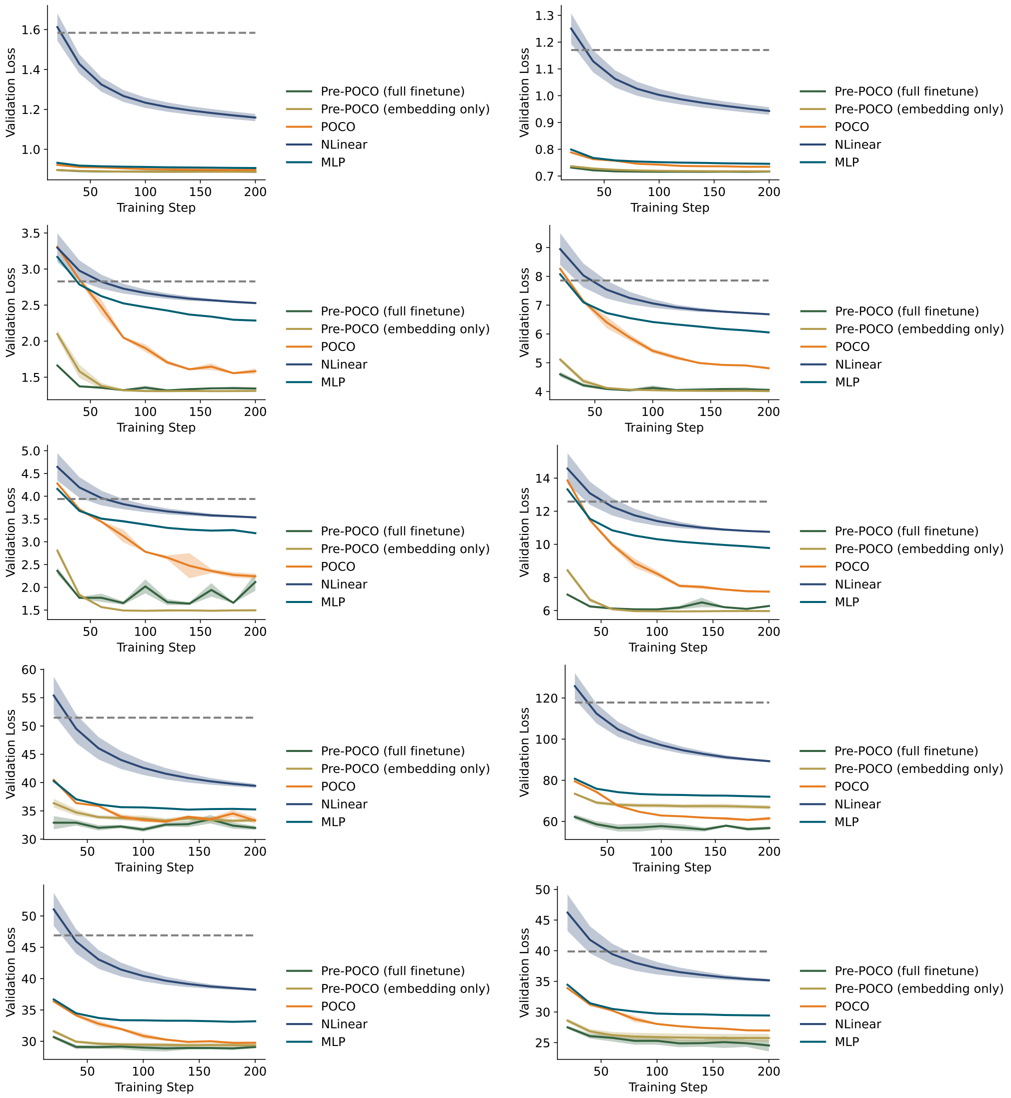

Figure S12: **Fine-tuned POCO rapidly adapts to new sessions with minimal updates.** Validation loss over 200 training steps for various fine-tuning strategies across 10 sessions. The top row contains the results of 2 sessions in the mice dataset. The second and third rows contain results of 4 sessions for Deisseroth's zebrafish dataset, where we are predicting the first 512 PCs. The last 2 rows contain results of 4 sessions for Ahren's zebrafish dataset, where we are also predicting the first 512 PCs. Error shades: SEM across 3 seeds.

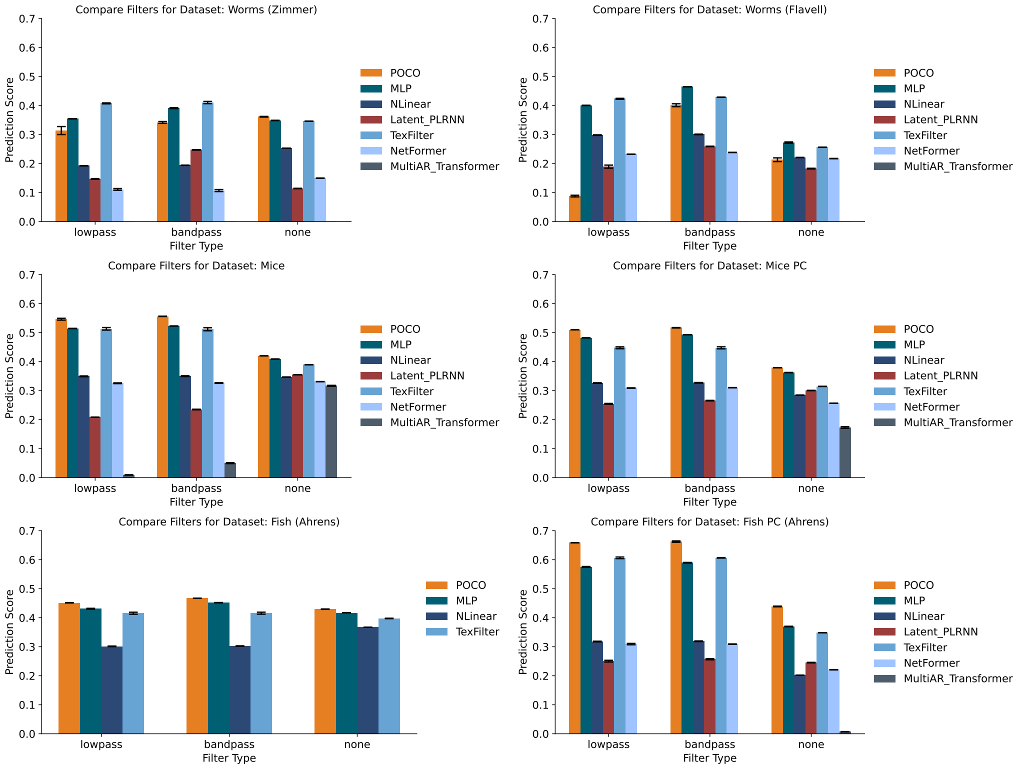

Figure S13: **Low-pass filtering improves POCO forecasting in most datasets.** Prediction score under different filtering regimes (none, low-pass, band-pass) across datasets. Cutoff frequency for low-pass filter is $0.1 \times f_s$, where f_s is the sampling frequency. A band-pass filter additionally removes frequency components lower than $5 \times 10^{-3} \times f_s$. Error bars: SEM over 2 seeds.

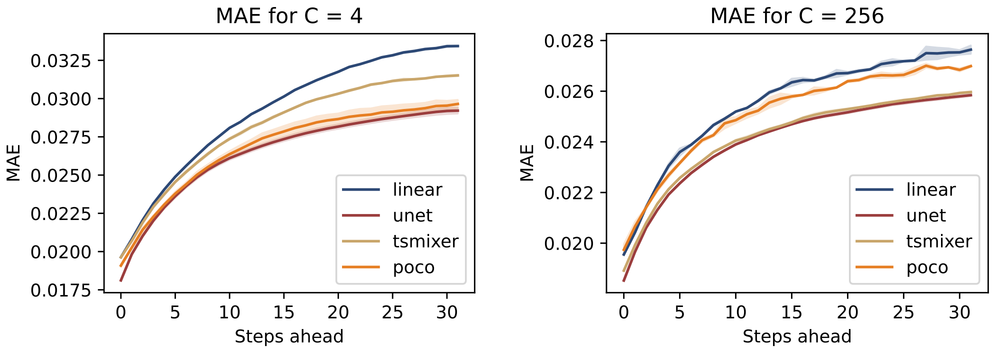

Figure S14: **POCO matches or outperforms volumetric models on Zapbench at short context.** Mean absolute error over 32 prediction steps on Zapbench with $C = 4$ and $C = 256$. POCO matches UNet at short context but underperforms with longer context. Performance data for models other than POCO are from [32].

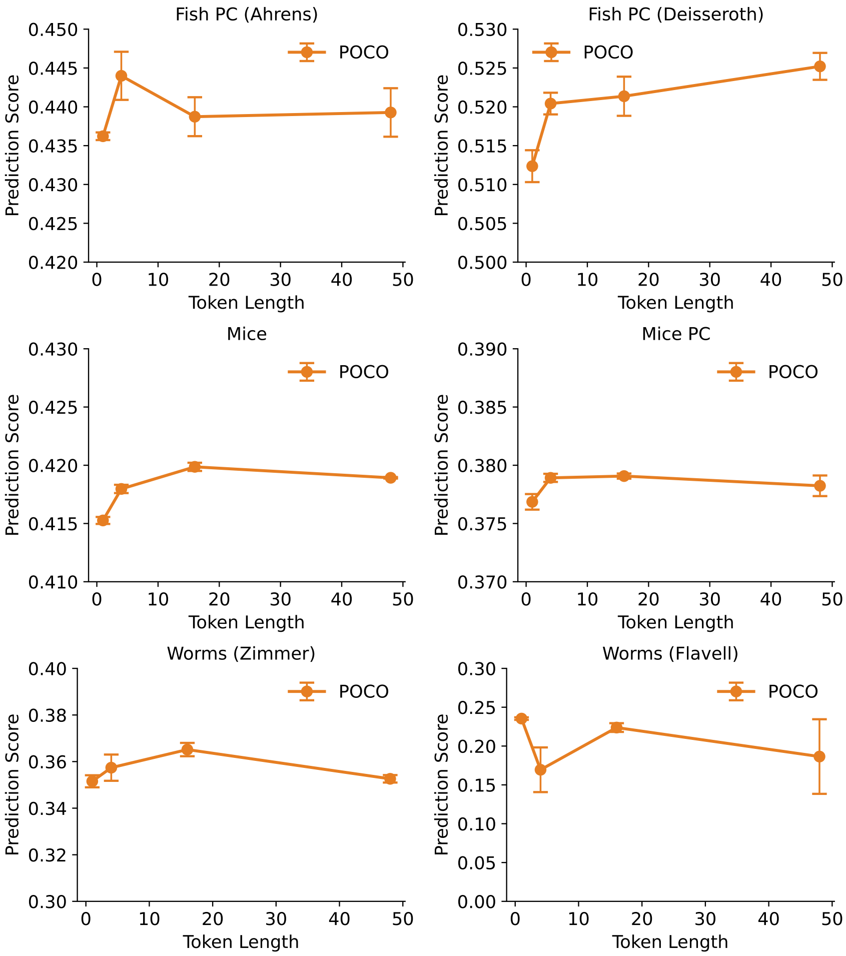

Figure S15: **Token length T_C influences POCO performance across datasets.** Using $T_C = 1$ is suboptimal and computationally expensive. $T_C = 16$ balances performance and cost. Results averaged over 3 seeds.

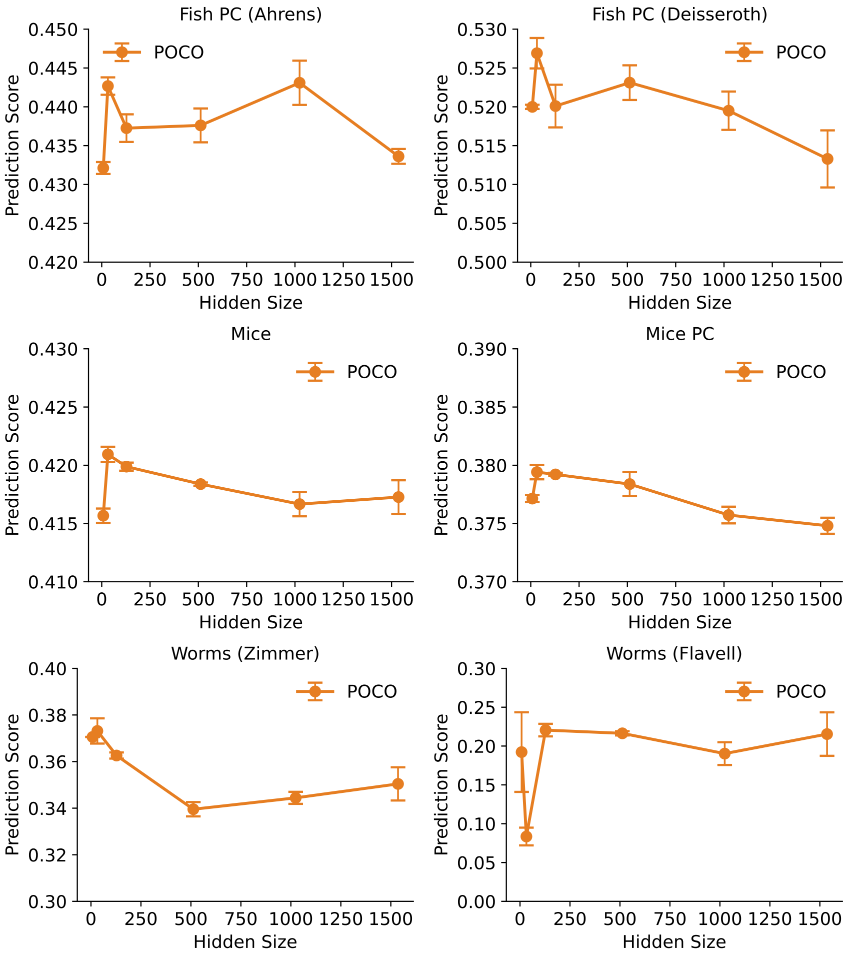

Figure S16: **Embedding size moderately affects POCO performance.** Validation performance shown across 6 datasets as a function of embedding dimension d. Default value $d = 128$ achieves near-peak performance. SEM across 3 seeds.

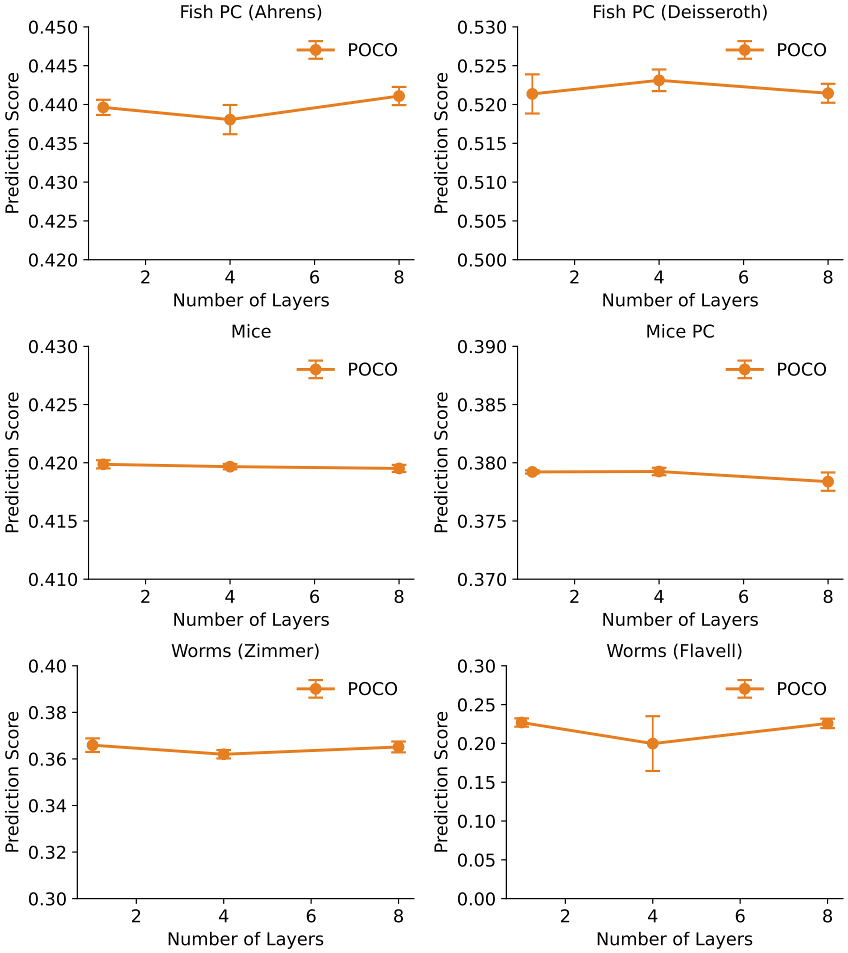

Figure S17: **POCO performs robustly across number of encoder layers.** Increasing Perceiver-IO layers from 1 to 8 shows minimal gain, suggesting 1 layer suffices for most datasets. Error bars: SEM across 3 seeds.

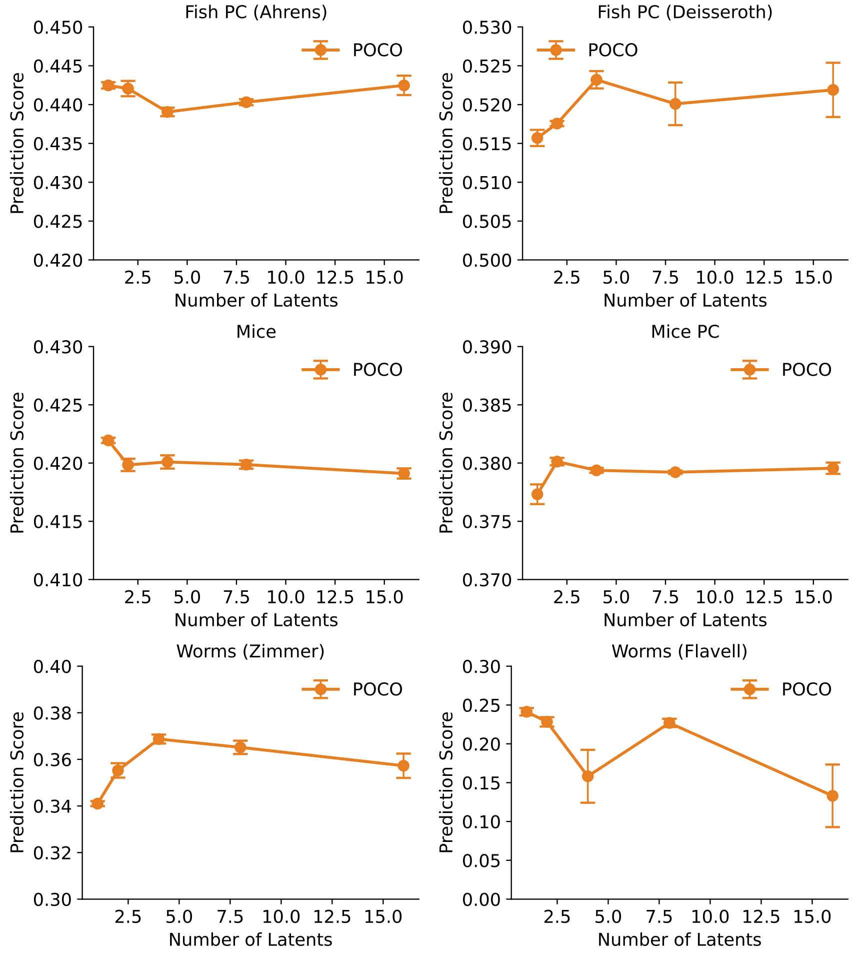

Figure S18: **Number of latents affects POCO performance marginally.** Validation score shown for different latent counts in the encoder. Performance saturates near 8 latents. SEM across 3 seeds.

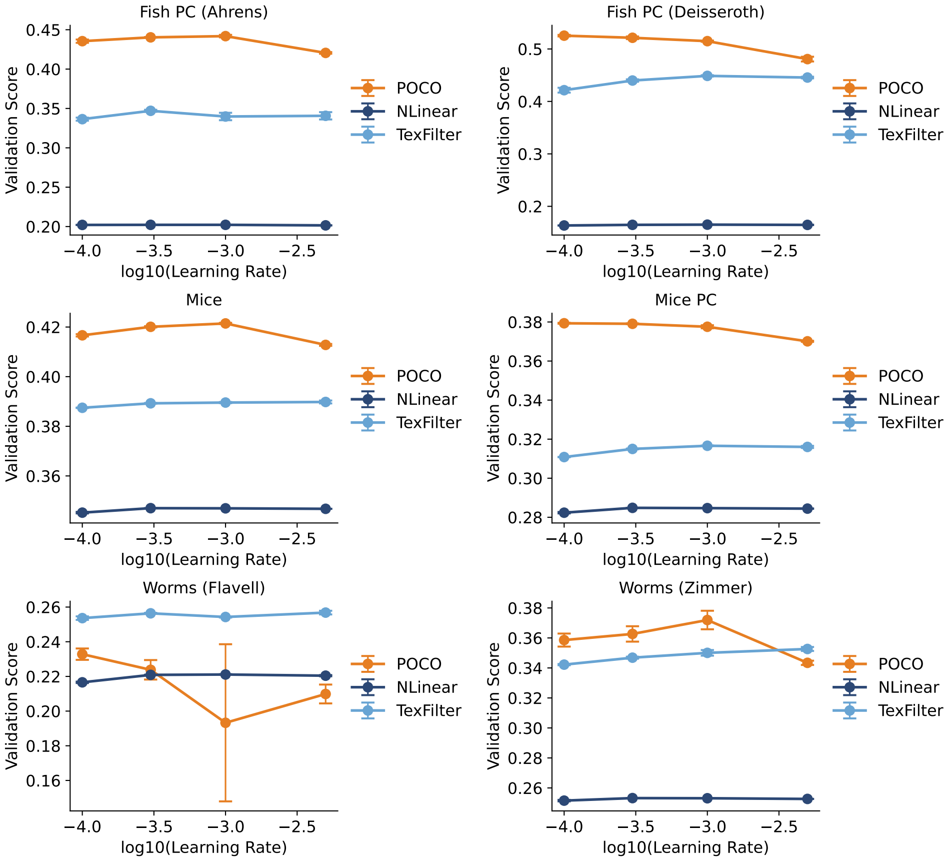

Figure S19: **POCO is robust to a range of learning rates.** Validation scores for different learning rates across datasets. Best performance usually occurs near 0.0003. SEM across 3 seeds.

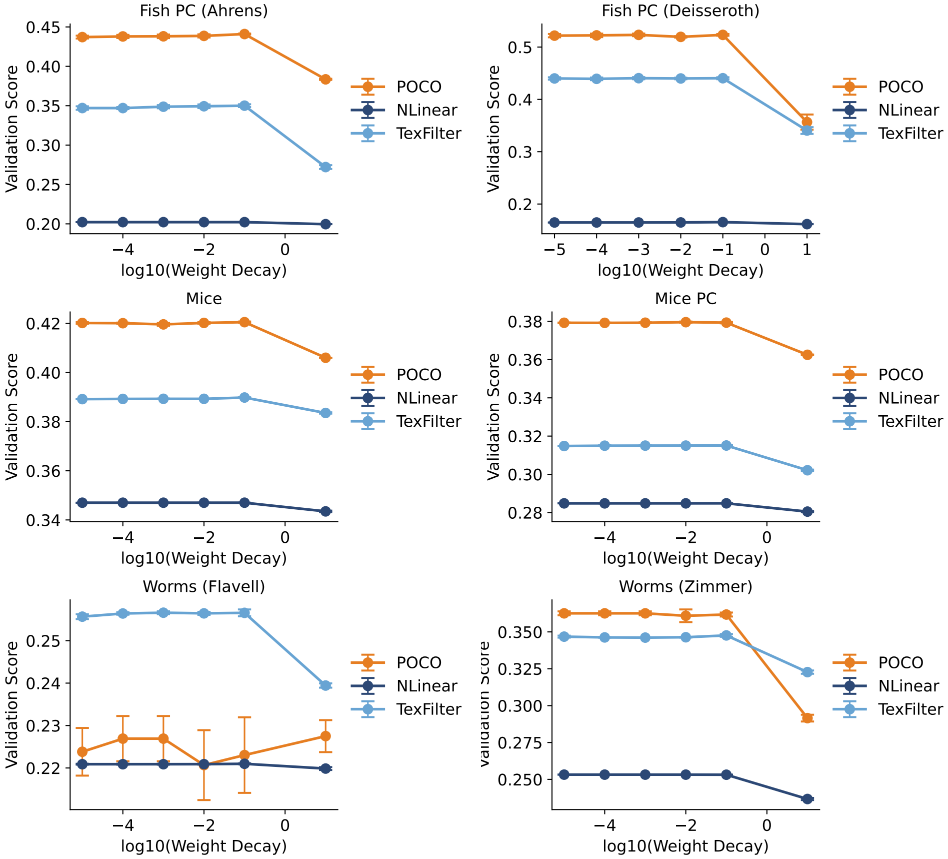

Figure S20: **POCO is relatively insensitive to weight decay settings.** Prediction score across 6 datasets with varied weight decay values. Performance is stable over several orders of magnitude. SEM across 3 seeds.

B Datasets

B.1 Dataset Details

Here we summarize the datasets used in this work. Additional details on the datasets can be found in the original dataset publications.

Zebrafish (Deisseroth). Parts of the dataset are published in [1]. Larval zebrafish expressing nuclear-localized GCaMP6s were used for two-photon calcium imaging. Fish were head-fixed for whole-brain imaging while retaining the ability to perform tail movements. Neural activity was recorded at about 1 Hz, yielding volumetric data from 8,000–22,000 neurons per fish. The imaging data were motion-corrected, segmented into nuclei, and fluorescence traces were extracted and converted into $\Delta F/F$. ROIs were assigned brain region labels based on anatomical landmarks.

Experiments were conducted across four cohorts. The Spontaneous cohort was imaged during natural, unstimulated behavior. The Shocked cohort received randomly timed mild electric shocks during imaging. The Reshocked cohort was pre-exposed to a behavioral challenge before imaging, then imaged under the same shock protocol. The Ketamine cohort received ketamine exposure prior to shock delivery during imaging. There are 5 fish for each cohort, except that the Ketamine cohort has 4 fish. These conditions allowed us to examine brain-wide neural dynamics under varying levels of behavioral and pharmacological perturbation.

Zebrafish (Ahrens). The dataset is published in [11]. A light-sheet imaging setup is used to record whole-brain neural activity from larval zebrafish expressing nuclear-localized GCaMP6f. Imaging was performed at ~ 2.1 Hz for ~ 50 minutes across 18 fish, resulting in $\sim 6,800$ time points and $\sim 80,000$ segmented neurons per animal. Subject 8, 9, and 11 are excluded due to file incompatibility issues, thus, only 15 fish are used. During imaging, fish were exposed to a battery of visual stimuli, including phototaxis cues, moving gratings (optomotor response), looming discs (escape), dark flashes, and spontaneous illumination blocks. Although behavioral data were also collected, we used only the neural activity traces for modeling, and no stimulus or behavioral labels were provided to the model.

Mice (Harvey). The dataset is partly described in [40], and the mesoscopic calcium imaging technique is detailed in [3]. It includes multi-session two-photon calcium imaging recordings from layer 2/3 neurons in four cortical regions: primary visual cortex (V1), secondary motor cortex (M2), posterior parietal cortex (PPC), and retrosplenial cortex (RSC). Mice expressing GCaMP6s were head-fixed and allowed to run spontaneously on an air-supported spherical treadmill in complete darkness. Imaging was performed using a large field-of-view two-photon microscope, capturing neural activity at about 5.4 Hz from two planes per region. Data were collected across multiple sessions and animals, with each session lasting 45–60 minutes. Four mice were recorded, with 1, 5, 4, and 2 sessions respectively. For the first two mice, all four regions were recorded. For the third mouse, two sessions included simultaneous recordings of PPC, RSC, and V1, while the other two sessions included PPC, RSC, and M2. For the fourth mouse, only PPC, RSC, and V1 were recorded. Motion correction and ROI extraction were performed using standard pipelines. Note that no behavioral or anatomical labels were provided to the model.

***C. elegans* (Zimmer).** The dataset is described in [23]. Code for data loading and preprocessing is partly based on [25]. This dataset contains whole-brain calcium imaging recordings from *C. elegans*, using a pan-neuronally expressed nuclear calcium indicator GCamp5K. Neural activity was recorded from 5 animals at 2.85 volumes per second for 18 minutes per animal. The imaging volume covered the head ganglia, including most sensory neurons, interneurons, all head motor neurons, and the anterior ventral cord motor neurons. Each recording included 107–131 neurons, with most active neurons identifiable by cell class. Animals were recorded either under constant oxygen or during alternating oxygen stimuli. Only neural activity traces were used in our experiments; stimulus and cell identity labels were not provided to the model.

***C. elegans* (Flavell).** The dataset is published in [4]. This dataset includes brain-wide calcium imaging recordings from freely moving *C. elegans* using a strain expressing nuclear-localized GCaMP7f and mNeptune2.5 in all neurons. Recordings were conducted on a custom dual-light-path microscope with closed-loop tracking to maintain the worm's head within the imaging field. Neural signals were extracted using automated segmentation and tracking based on 3D U-Net and registration pipelines. We used only the 40 sessions with NeuroPAL neuron identity labels—21 recorded under baseline

conditions and 19 with a noxious heat stimulus that induced a persistent behavioral state change. Only the neural activity traces were used in our experiments; stimulus timing and neuron identity labels were not provided to the model.

Zapbench. The benchmark is described in [32]. This dataset consists of whole-brain calcium imaging from a single head-fixed larval zebrafish recorded during fictive behavior in a virtual reality environment. Over the course of a two-hour session, the fish was exposed to nine structured visual stimulus conditions designed to elicit a range of sensorimotor responses. Neural activity was recorded at cellular resolution using light-sheet fluorescence microscopy, resulting in a 4D volumetric dataset spanning 71,721 segmented neurons. Postprocessing included motion correction, custom elastic alignment, and manual neuron segmentation using a Flood-Filling Network. Only the extracted per-neuron calcium traces were used in our experiments; stimulus information was not provided to the model.

B.2 Data Processing

For Zapbench, we follow the code provided with the benchmark to directly load the processed data [32]. For other datasets, we begin by z-scoring the raw fluorescence values for each neuron so that each calcium trace has zero mean and unit variance. This normalization ensures that all neurons are equally weighted in the model—an active neuron with a high baseline activity contributes similarly to a less active one. For Deisseroth's zebrafish dataset, where raw calcium traces are unavailable, we instead apply z-scoring to the denoised activity extracted using constrained nonnegative matrix factorization (CNMF) [41]. For experiments involving frequency filtering, we apply a zero-phase fourth-order Butterworth filter. The default low-pass filter removes frequency components above $0.1 \times f_s$, where f_s is the sampling frequency. A band-pass filter additionally removes components below $5 \times 10^{-3} \times f_s$. After filtering, we optionally reduce dimensionality using principal component analysis (PCA), applied to the z-scored (and optionally filtered) neural activity matrix. The magnitudes of the resulting principal components are preserved, meaning that dominant components naturally carry more weight in the loss function.

B.3 Data Partitioning

We first divide each session into segments of 1,000 time steps. Each segment is then split into training, validation, and test partitions using a 3:1:1 ratio. The final segment of a session may contain more than 1,000 steps to maximize use of the available data. To construct the training set, we extract all possible consecutive subsequences of length $C + P$ (64 by default) from the training partition using a sliding window with stride 1. For example, if a session contains 2,500 steps, the training partitions would cover steps $[1, 600]$ and $[1001, 1900]$, and the training subsequences would include $\mathbf{x}_{1:1+C+P}, \mathbf{x}_{2:2+C+P}, \dots, \mathbf{x}_{600-C-P+1:600}$ and similarly for the second partition. Validation and test sets are constructed in the same way. However, because evaluation is computationally expensive for single-cell resolution zebrafish datasets, we use a larger stride when extracting subsequences from the validation set: stride 32 for Ahrens' dataset and stride 8 for Deisseroth's dataset. Note that striding is applied only to the validation set in the context of single-cell level prediction.

For Zapbench, we instead use the provided tools to load the dataset [32]. One notable difference is that in Zapbench, the dataset is first divided by the stimulus condition then divide each condition into train, validation and test sets.

C Additional Model Details

C.1 POCO

In POCO, we use Rotary Position Embeddings (RoPE) in all attention layers [45]. In RoPE, a timestamp t is assigned to each query token $\mathbf{q}_i$ and key token $\mathbf{k}_j$, and the attention score is computed as

$$\mathbf{a}_{ij} = \text{softmax}\left((\mathbf{R}(t_i)\,\mathbf{q}_i)^T (\mathbf{R}(t_j)\,\mathbf{k}_j) \right), \quad (8)$$

where $\mathbf{R}(t)$ is a time-dependent rotation matrix. We follow POYO [5] to construct $\mathbf{R}(t)$, defined as a composition of 2×2 rotation matrices with periods T_i logarithmically spaced between $T_{\min}$ and $T_{\max}$. In our experiments, we use $T_{\min} = 10^{-3} \times T_{\max}$ and set $T_{\max} = 100$ by default.

We also need to define the timestamps. For the first attention layer,

$$\mathbf{L}_1 = \text{Attention}_0(Q = \mathbf{L}_0;\, K, V = \mathbf{E}) \in \mathbb{R}^{N_L \times d}, \tag{9}$$

we assign timestamps to the trace tokens $\mathbf{E}(i, k)$ as $t_{i,k} = (k-1)T_C$, which corresponds to the starting timestep of the token. For the latent tokens $\mathbf{L}_0(j)$, we set

$$t_j = \frac{j-1}{N_L} C, \quad j \in [N_L], \tag{10}$$

so that the timestamps uniformly span the input context length C. Timestamp for latents in later attention layers $\mathbf{L}_l$ are defined in the same way. For the final layer, the query tokens are given a constant time step $t = C$. Intuitively, the latents can be viewed as encoding the global population state at different moments in the past, and in the final layer, we query how those past states influence the future.

For Zapbench experiments, we modify these parameters to accommodate shorter or longer context lengths: specifically, we use $T_{\max} = 50$, $T_C = 4$ for $C = 4$, and $T_{\max} = 400$, $T_C = 64$ for $C = 256$. The default token length is $T_C = 16$, resulting in 3 tokens per neuron when $C = 48$.

For the conditioning layer, we initialize the weights $\mathbf{W}_\beta, \mathbf{W}_\gamma$ and biases $\mathbf{b}_\beta, \mathbf{b}_\gamma$ of the linear mappings (from the final attention layer output to FiLM conditioning parameters) to zero, ensuring that POCO produces a flat output at initialization. We follow POYO on additional model details: We use an additional feedforward layer following each attention layer. Residual connections are used for both the attention layers and the feedforward layers that follow. We use 1 attention head for the first and last attention layer, and 8 heads for intermediate self-attention layers. We set the embedding and latent size to $d = 128$; in rotary attention, each dimension of each head is 64. We use a dropout of 0.2 in the feed-forward layers following the attention layers and a dropout of 0.4 on the output of intermediate self-attention layers and the accompanying feedforward layers. We initialize the embeddings from $\mathcal{N}(0, \sigma_0^2)$ with $\sigma_0 = 0.02$.

C.2 Ablation Study

In the ablation study, we considered several alternative architectures. First, to directly use the POYO model to generate 16-steps prediction, we use a linear projection on top of the output of the last layer

$$\begin{aligned} \mathbf{L}_{L+2} &= \text{Attention}_{L+1}(Q = \mathbf{U}_j; K, V = \mathbf{L}_{L+1}) \in \mathbb{R}^{N_j \times d}, \\ \hat{\mathbf{x}}_{t:t+P} &= \mathbf{W}_{out}\mathbf{L}_{L+2}^T + \mathbf{b}_{out}, \end{aligned} \tag{11}$$

where $\mathbf{W}_{out} \in \mathbb{R}^{P \times d}$.

Another alternative we tested is replacing the population encoder with a univariate Transformer, which takes the calcium trace of a single neuron instead of encoding the whole population. Specifically, for each neuron i, we still use $T_C = 16$ steps to form one token, but the token embedding no longer involves the unit and session embedding:

$$E(k) = \mathbf{W} x^{(j)}_{r_k - T_C : r_k, i} + \mathbf{b}. \tag{12}$$

We then apply a standard 1-layer transformer encoder used in recent time-series forecasting work [47]. Note that the Transformer is applied separately to each neuron. We concatenate hidden states of the last encoder layer, resulting in a $dC/T_C = 128 \times 3$-dimensional vector for each neuron. Finally, we generate 16-step prediction by applying a linear projection on the concatenated hidden state.

C.3 Other Baselines

NLinear. In NLinear [55], the last step of the input context is subtracted from the input sequence, and the residual is passed through a linear layer to generate predictions ($\mathbb{R}^C \to \mathbb{R}^P$). The last step is then added back to the output. This design is effective in time series forecasting (TSF) tasks as it helps address distributional shifts between training and test sets. NLinear is a univariate model—each neuron's prediction depends only on its own history—which also makes it naturally capable of processing multi-session data, as it can handle an arbitrary number of neurons.

DLinear. We adopt the implementation from [55]. DLinear first decomposes each time series into a trend and a seasonal component. The trend component is obtained by applying a moving average filter

with kernel size $2\lfloor C/4 \rfloor + 1$, and the seasonal component is the residual obtained by subtracting the trend from the original input. Both components are predicted independently using linear projections, and the final output is their sum. Like NLinear, DLinear is also univariate.

MLP. We use a two-layer MLP with ReLU activation and hidden size 1024. This model is equivalent to POCO without FiLM conditioning. It is also univariate.

We include PLRNN as a baseline due to its demonstrated ability to reconstruct chaotic dynamical systems. We use the implementation from [7]. The model defines a dynamical system over a d-dimensional latent state vector $\mathbf{z}_t$ as:

$$\mathbf{z}_t = \mathbf{A}\mathbf{z}_{t-1} + \mathbf{W}\,\phi(\mathbf{z}_{t-1}) + \mathbf{h},$$

where $\mathbf{A} \in \mathbb{R}^{d\times d}$ is a diagonal matrix encoding self-connections, $\mathbf{W} \in \mathbb{R}^{d\times d}$ contains off-diagonal weights for inter-unit interactions, and $\mathbf{h} \in \mathbb{R}^d$ is a bias term. The activation function ϕ is ReLU. We set $d = 512$. In our setup, due to the slow dynamics of calcium traces, we use the alternative formulation

$$\mathbf{z}_t = \alpha\,(\mathbf{A}\mathbf{z}_{t-1} + \mathbf{W}\,\phi(\mathbf{z}_{t-1}) + \mathbf{h}) + (1-\alpha)\mathbf{z}_{t-1},$$

where $\alpha = 0.05$. To generate predictions, we first map the last step from the context to the latent state:

$$\mathbf{z}_C = \mathbf{W}_{\text{in}}\mathbf{x}_C + \mathbf{b}_{\text{in}},$$

then evolve the latent state for $P = 16$ steps and map it back to the observation space:

$$\hat{\mathbf{x}}_{C+t} = \mathbf{W}_{\text{in}}^{\dagger}(\mathbf{z}_{C+t} - \mathbf{b}_{\text{in}}), \quad t \in [P],$$

where $\mathbf{W}_{\text{in}}^{\dagger}$ denotes the pseudo-inverse of $\mathbf{W}_{\text{in}}$. PLRNN is multivariate, but it only uses the last time step of the context to generate prediction. To fully utilize the input during training, we instead initialize the latent state $\mathbf{z}_1$ from $\mathbf{x}_1$ and evolve the latent dynamics model for $C + P - 1$ steps. We apply sparse teacher forcing (STF) [35], injecting the ground-truth state every four steps to reduce the drift from the groundtruth for effective training; STF is disabled during the final P prediction steps to simulate free prediction. For multi-session training, the latent dynamics (PLRNN parameters) are shared across sessions, while the input mapping $\mathbf{W}_{\text{in}}$ is session-specific to capture individual variability.

AR_Transformer. We also include an autoregressive Transformer [50] as a baseline to assess the performance of classical autoregressive architectures in neural population modeling. A linear layer ($\mathbb{R}^N \to \mathbb{R}^d$) maps each time step's population activity to a d-dimensional embedding, producing C tokens. Here we use $d = 512$. These tokens are passed through a 4-layer Transformer with causal attention masks. A final linear projection ($\mathbb{R}^d \to \mathbb{R}^N$) maps the output embeddings to next-step predictions. To forecast $P = 16$ steps, the model is applied autoregressively: at each step, the predicted activity is appended and used as input for the next prediction. As with PLRNN, for multi-session training, the Transformer backbone is shared, while the input and output projection layers are session-specific.

TSMixer. TSMixer [16] is an all-MLP architecture for time series forecasting. It consists of stacked mixer blocks, each comprising a time mixer (a linear projection operating along the time dimension) and a feature mixer (an MLP with hidden size 64 that operates across the neuron dimension). The inclusion of the feature mixer makes TSMixer a multivariate model. We use the default hyperparameter settings: two mixer blocks and a dropout rate of 0.1.

TexFilter. TexFilter [54] is a recent MLP-based method that incorporates frequency-domain filtering. It applies a context-dependent filter on the Fourier-transformed input, allowing the model to selectively enhance or suppress specific frequency components. In addition to the MLP layer, TexFilter uses learnable complex embeddings that modulate the frequency representation. TexFilter is univariate. In our setup, we set the embedding size to 128 and initialize the embeddings from $\mathcal{N}(0, \sigma_0^2)$, $\sigma_0 = 0.02$, with a dropout rate of 0.3.

TCN. ModernTCN [33] employs modernized convolutional blocks for time series modeling. The input is first divided into patches of size $P = 4$, which are embedded into $D = 64$-dimensional vectors. These embeddings are processed using convolutional blocks that capture both temporal and cross-neuron dependencies, followed by a linear readout for prediction. This makes ModernTCN a multivariate model. We follow the original work's hyperparameters for forecasting, except for the

convolutional kernel sizes, which are adjusted to 17 (large) and 5 (small) to better suit our shorter context window.

Netformer. Netformer [31] is a recent approach for modeling dynamical connectivity in neural population activity. It embeds each neuron's past activity trace and uses an attention layer to compute interaction weights A across neurons. The next-step prediction is given by:

$$\hat{\mathbf{x}}_{t+1} = A\mathbf{x}_t + \mathbf{x}_t.$$

Although the original work only evaluated next-step prediction, we extend it to multi-step forecasting by recursively applying the same update. However, we observed instability when training on long horizons and therefore applied a softmax to the attention weights to stabilize learning. Furthermore, to further improve stability, we also applied an instance normalization layer on the input and later unnormalized the prediction as in recent TSF works [54, 47]. As in the original Netformer, we apply layer normalization along the time dimension, and use an embedding size of 30. Netformer is a multivariate model but is naturally compatible with multi-session training, as the attention mechanism is independent of the number of neurons.

D Experiments

D.1 Training

For multi-session training, we train each model for 10^4 steps. Single-session models are instead trained for 5×10^3 steps considering the smaller training set size. For all models, we used AdamW [30] optimizer with learning rate 0.0003 and weight decay 10^{-4}. At each training step, a batch of 64 sequences is sampled from the training sets of all sessions. For single-cell prediction in larval zebrafish, we reduce the batch size to 8 for Deisseroth's zebrafish dataset and to 4 for Ahrens' zebrafish dataset due to memory constraints. Gradient clipping is applied to limit the gradient norm to 5. Most models are trained using mean squared error (MSE) loss averaged over the P prediction steps. However, for PLRNN, the autoregressive Transformer, and Netformer—models that generate one-step predictions—the loss is computed across all $C + P - 1$ steps. Models are evaluated on the validation set every 100 training steps, and we report test-set performance using the checkpoint with the lowest validation loss.

D.2 Simulation

Code for simulation is adapted from [40]. We generate synthetic neural data from a chaotic RNN with a $\tanh$ nonlinearity, governed by the following dynamical equation:

$$\mathbf{r} = \tanh(\mathbf{h}), \quad \tau \frac{d\mathbf{h}}{dt} = -\mathbf{h} + g\mathbf{J}\mathbf{r} + \sqrt{2\tau\sigma^2}\boldsymbol{\xi}, \quad \mathbf{h}(0) \sim \mathcal{U}[-1, 1],$$

where $\mathbf{h}$ is the internal (pre-activation) state, $\mathbf{r} = \tanh(\mathbf{h})$ is the firing rate, $\mathbf{J}$ is a recurrent weight matrix, $g = 2.0$ is a gain factor, $\boldsymbol{\xi}$ are N independent Gaussian white noise processes with zero mean and unit variance, $\sigma = 0.1$ controls the noise variance, and $\tau = 0.1$ is the time constant. We discretize the dynamics using Euler's method with time step $\Delta t = 0.01$:

$$\mathbf{h}_{t+1} = \mathbf{h}_t + \frac{\Delta t}{\tau}\left(-\mathbf{h}_t + g\mathbf{J}\tanh(\mathbf{h}_t)\right) + \sqrt{2\sigma^2 \frac{\Delta t}{\tau}}\, \boldsymbol{\xi}_t, \quad \mathbf{r}_t = \tanh(\mathbf{h}_t),$$

where $\boldsymbol{\xi}_t \sim \mathcal{N}(0, I)$ at each step. We z-score the firing rates $\mathbf{r}_t$, and to simulate the slower sampling rate of calcium imaging, we average $\mathbf{r}_t$ over every $f = 5$ time steps. The simulation runs from $t = 0$ to $t = 4096 \times f\tau$, producing 4096 time steps of synthetic data—comparable in length to real neural recordings.

D.3 Finetuning

For pretraining and finetuning experiments, we partition each dataset and use approximately 80% of the sessions for pretraining. Specifically, for Deisseroth's zebrafish dataset, we reserve the last session from each cohort for finetuning, yielding 15 sessions for pretraining. For Ahrens' zebrafish dataset, we finetune on the last 4 sessions and pretrain on the remaining 11. For the mice dataset from Harvey et al., we finetune using the 2 sessions from the last mouse. All models are finetuned for 2,000 steps and evaluated every 20 steps.

D.4 Unit Embedding

Although multi-session POCO learns a shared unit embedding space across sessions, the additional session embedding may introduce session-specific offsets. To avoid this confound, we analyze and visualize unit embeddings separately for each session. Let $U(i)$ denote the embedding of neuron i, and $S_u^{(j)}$ the set of neurons in region u from session j. The cosine similarity between regions u and v is computed as:

$$C(u,v) = \frac{1}{\sum_j |S_u^{(j)}||S_v^{(j)}|} \sum_j \sum_{i_u \in S_u^{(j)}} \sum_{i_v \in S_v^{(j)}} \frac{U(i_u)^T U(i_v)}{\|U(i_u)\| \|U(i_v)\|}, \quad \text{for } u \neq v,$$

$$C(u,u) = \frac{1}{\sum_j |S_u^{(j)}|(|S_u^{(j)}| - 1)} \sum_j \sum_{\substack{i_u, i_v \in S_u^{(j)} \\ i_u \neq i_v}} \frac{U(i_u)^T U(i_v)}{\|U(i_u)\| \|U(i_v)\|}.$$

To highlight relative similarity patterns, we compute a row-wise normalized similarity:

$$\bar{C}(u,v) = \frac{C(u,v) - \min_{v'} C(u,v')}{\max_{v'} C(u,v') - \min_{v'} C(u,v')}.$$

This normalized similarity more clearly reveals which brain regions are functionally closer or further from a given region u. The same analysis can be applied using other distance metrics, such as Euclidean distance.

D.5 Multi-Dataset Training

To support multi-dataset training, we assign a separate dataloader to each dataset. At each training step, we sample one batch from each dataset, compute the loss independently, and sum the losses before performing a single backward pass. To help POCO handle cross-dataset differences, we add a dataset-specific embedding to each input token:

$$E(i,k) = \mathbf{W} x^{(j)}_{r_k - T_C : r_k, i} + \mathbf{b} + \text{UnitEmbed}(i,j,u) + \text{SessionEmbed}(j,u) + \text{DatasetEmbed}(u),$$

where $u \in [D]$ is the dataset index and D is the total number of datasets.

D.6 Experiments on Zapbench

For Zapbench, we follow the provided script for dataloading and testing [32]. We train POCO for 25 epochs with a batch size of 8, validating after every epoch. We report test-set performance using the checkpoint with the lowest validation loss. Following the benchmark protocol, we use mean absolute error (MAE) as the loss function. The *TAXIS* condition is excluded during training, and performance is evaluated on the remaining stimulus conditions.

E Compute Resources

All models are trained on NVIDIA A100 or H100 GPUs paired with AMD EPYC 9454 CPUs. Only a single CPU thread is used throughout training. Data preprocessing for all datasets takes less than one CPU day in total, and generating all synthetic data requires under two CPU days.

For multi-session prediction of the first 512 principal components (PCs) from Deisseroth's zebrafish dataset, training POCO for 10^4 steps takes less than 30 minutes, including data loading and validation time. Despite using a high-end GPU, this training setup consumes under 4GB of GPU memory. Computational cost increases for datasets with more neurons: for single-cell prediction on Ahrens' zebrafish dataset, training consumes up to 48GB of GPU memory and completes in under one hour.

While training a single model is fast, some experiments involve training many models. For example, training a single-session model for each session in each dataset across 4 random seeds involves training over 300 models in total. The estimated total GPU time across all experiments is approximately 1,500 hours.

The same hardware setup (H100 GPU) is used to measure finetuning and inference speed. When finetuning POCO on the first 512 PCs of Deisseroth's dataset, we find that adapting the embedding

for 200 training steps takes less than 15 seconds, and forecasting a single sequence takes only 3.5 milliseconds.

NeurIPS Paper Checklist

1. **Claims**

   Question: Do the main claims made in the abstract and introduction accurately reflect the paper's contributions and scope?

   Answer: [Yes]

   Justification: All claims and stated contributions are supported by experiments.

2. **Limitations**

   Question: Does the paper discuss the limitations of the work performed by the authors?

   Answer: [Yes]

   Justification: Limitations of the current work are discussed at the end of the paper, in the Discussion section.

3. **Theory assumptions and proofs**

   Question: For each theoretical result, does the paper provide the full set of assumptions and a complete (and correct) proof?

   Answer: [NA]

   Justification: The paper does not include theoretical results.

4. **Experimental result reproducibility**

   Question: Does the paper fully disclose all the information needed to reproduce the main experimental results of the paper to the extent that it affects the main claims and/or conclusions of the paper (regardless of whether the code and data are provided or not)?

   Answer: [Yes]

   Justification: Details on model and experiments can be found in the Appendix. Codes and instructions on processing public datasets used in the paper will be made available on Github.

5. **Open access to data and code**

   Question: Does the paper provide open access to the data and code, with sufficient instructions to faithfully reproduce the main experimental results, as described in supplemental material?

   Answer: [Yes]

   Justification: We use a mix of publicly available and collaboration-only datasets. Three datasets (Ahrens zebrafish, Zimmer and Flavell C. elegans) are publicly available; the remaining two (Deisseroth zebrafish and Harvey mouse) are available upon request or under collaboration agreements. Code to reproduce all results and instructions for accessing public datasets will be released on GitHub.

6. **Experimental setting/details**

   Question: Does the paper specify all the training and test details (e.g., data splits, hyperparameters, how they were chosen, type of optimizer, etc.) necessary to understand the results?

   Answer: [Yes]

   Justification: Details of experiments are specified in the Appendix.

7. **Experiment statistical significance**

   Question: Does the paper report error bars suitably and correctly defined or other appropriate information about the statistical significance of the experiments?

   Answer: [Yes]

   Justification: We included error bars for all applicable plots, where we mostly use standard error of the mean (SEM) as stated in the caption of each figure. For tables, we include 95% confidence intervals, but the Normality of errors is not verified.

8. **Experiments compute resources**

Question: For each experiment, does the paper provide sufficient information on the computer resources (type of compute workers, memory, time of execution) needed to reproduce the experiments?

Answer: [Yes]

Justification: We provide the amount of compute used and the approximate run time for all experiments in the Appendix.

9. **Code of ethics**

   Question: Does the research conducted in the paper conform, in every respect, with the NeurIPS Code of Ethics `https://neurips.cc/public/EthicsGuidelines`?

   Answer: [Yes]

   Justification: We make sure the work conforms with the NeurIPS Code of Ethics.

10. **Broader impacts**

    Question: Does the paper discuss both potential positive societal impacts and negative societal impacts of the work performed?

    Answer: [Yes]

    Justification: While this work focuses on modeling spontaneous neural activity in animal datasets, the modeling approach introduced here—particularly POCO's ability to generalize across individuals and support rapid adaptation—could inform future neurotechnologies such as brain-computer interfaces, closed-loop control systems, and real-time neuroprosthetics. As such applications emerge, considerations around safety, robustness, and clinically useful deployment will become increasingly relevant.

11. **Safeguards**

    Question: Does the paper describe safeguards that have been put in place for responsible release of data or models that have a high risk for misuse (e.g., pretrained language models, image generators, or scraped datasets)?

    Answer: [NA]

    Justification: The paper does not release data or models with high risks of misuse.

12. **Licenses for existing assets**

    Question: Are the creators or original owners of assets (e.g., code, data, models), used in the paper, properly credited and are the license and terms of use explicitly mentioned and properly respected?

    Answer: [Yes]

    Justification: We use existing datasets and models with proper citation. Public datasets are cited in the manuscript with corresponding references. Where licenses are available (e.g., Creative Commons), we follow them; for datasets not publicly licensed, access was obtained via collaboration and all terms of use have been respected.

13. **New assets**

    Question: Are new assets introduced in the paper well documented and is the documentation provided alongside the assets?

    Answer: [Yes]

    Justification: Key usage and functions will be documented in the released code.

14. **Crowdsourcing and research with human subjects**

    Question: For crowdsourcing experiments and research with human subjects, does the paper include the full text of instructions given to participants and screenshots, if applicable, as well as details about compensation (if any)?

    Answer: [NA]

    Justification: The paper does not involve crowdsourcing nor research with human subjects.

15. **Institutional review board (IRB) approvals or equivalent for research with human subjects**

Question: Does the paper describe potential risks incurred by study participants, whether such risks were disclosed to the subjects, and whether Institutional Review Board (IRB) approvals (or an equivalent approval/review based on the requirements of your country or institution) were obtained?

Answer: [NA]

Justification: The paper does not involve crowdsourcing nor research with human subjects.

16. **Declaration of LLM usage**

Question: Does the paper describe the usage of LLMs if it is an important, original, or non-standard component of the core methods in this research? Note that if the LLM is used only for writing, editing, or formatting purposes and does not impact the core methodology, scientific rigorousness, or originality of the research, declaration is not required.

Answer: [NA]

Justification: Core method development does not involve LLMs; they are only used as coding and writing aid.
